# The long-term stability of solid-state oral pharmaceuticals exposed to simulated intravehicular space radiation

**DOI:** 10.1038/s41526-025-00469-w

**Published:** 2025-05-17

**Authors:** Ianik Plante, Vernie Daniels, Millennia Young, Ramona Gaza, Honglu Wu, John F. Reichard

**Affiliations:** 1 KBR, Houston, TX USA; 2 Statistics and Data Science, NASA Johnson Space Center, Houston, TX USA; 3 Leidos, Space Exploration and Mission Operations, Houston, TX USA; 4 Biomedical Research and Environmental Sciences Division, NASA Johnson Space Center, Houston, TX USA; 5 Department of Environmental and Public Health Sciences, University of Cincinnati, Cincinnati, OH USA

**Keywords:** Occupational health, Preventive medicine

## Abstract

Pharmaceutical products brought for space missions must remain effective and safe throughout the mission. Previous NASA experiments suggest that radiation exposure could threaten drug stability during long-duration space missions. The Exploration Medical Capability (ExMC) Element has evaluated this possibility by exposing four medications to simulated Galactic Cosmic Radiation (GCRSim) at the NASA Space Radiation Laboratory followed by a three-year storage period. The solid oral drug products Acetaminophen, Amoxicillin, Ibuprofen, and Promethazine were used. Identical lots of each medication were assigned to four experimental groups: the non-irradiated Johnson Space Center control group, the non-irradiated traveling control group, the irradiation group I (GRSim, 0.5 Gy), and the irradiation group II (GCRSim, 1.0 Gy). Drug products were assessed for active pharmaceutical ingredient, degradation impurities, and dissolution 2, 18, and 34 months after irradiation. All samples show comparable degradation, revealing that GCR exposure does not facilitate the degradation of the drugs.

## Introduction

The National Aeronautics and Space Administration (NASA) and its international partners are developing capabilities to conduct exploratory space missions beyond the Earth orbit. A roundtrip mission to Mars is expected to last two to three years^1^. The planetary exploration missions, unlike those to the International Space Station (ISS), will be too distant for resupply. Therefore, future exploration-class missions will need to be Earth-independent, notably for the supply of pharmaceutical products. Pharmaceuticals are used during space missions to treat or prevent medical conditions, including those occurring in microgravity, as well as potential injuries and illnesses. The pharmaceuticals brought for exploration space missions must remain safe and effective for the duration of the mission.

A drug product is a finished dosage form that contains a drug substance, the active pharmaceutical ingredient (API), in combination with other pharmacologically inactive ingredients known as excipients. The API gives the medication its pharmacological activity, while excipients are important for the manufacturing process and give the medication its physical characteristics. The word “drug” is used in this text as shorthand for a formulated drug product.

Drugs undergo physical and chemical degradation over time. Physical degradation affects characteristics such as appearance, hardness, or dissolution rate of the drugs. The dissolution rate is particularly important because it regulates the release of API from solid dosage forms, and—therefore—the rate and extent of absorption. Chemical degradation results from chemical reactions of any ingredient in the drug formulation, including the API, excipients, and impurities^2,3^. Environmental factors, such as moisture (i.e., relative humidity) and oxygen, are common co-reactants that enable hydrolysis and oxidation reactions that may contribute to degradation reactions. Chemical degradation decreases the API content and can reduce the therapeutic efficacy of medications. These reactions also form degradation impurities that accumulate over time and, if present at sufficiently high levels, can produce a risk for adverse health effects.

There is an existing concern that continuous exposure to ionizing radiation during long-duration space missions could compromise the stability of some drugs^4–7^ and, similarly, to loss of nutrients in food^8^. For drugs, this concern is driven by 1) a pilot study of lot-matched drug products suggesting increased degradation of drugs flown in space for up to 880 days^9^ and 2) terrestrial radiostability studies of individual drug products and substances exposed to high-dose ionizing radiation^10–17^. NASA-supported drug stability studies yield an inconsistent and incomplete picture of spaceflight drug stability. Several unpublished anecdotal studies suggest that the overall effect of long-duration space flight on API content of many drugs is minimal at most^9,18–20^. These studies have important design limitations that make quantitative comparisons across studies difficult^21^. Also, most spaceflight drug stability studies have focused on solid dosage forms (tablets or capsules). Only the study of Du et al. (2014), which included a subset of non-solid formulations, has shown that spaceflight reduces drug stability^9^.

Ionizing radiation produces both excited molecules and radicals in drug products, which are localized along radiation tracks^22^. Radicals can be produced directly and through the rupture of chemical bonds. Large radicals remain trapped within the matrix of the drug (so called “cage effect”) and undergo immediate germinate termination reactions^23–25^. Smaller species, such as the hydrogen atoms and electrons can diffuse through the matrix, although electrons may be trapped at some preferred sites. Radicals that escape combination or disproportionation typically undergo very slow radical decay, depending on the nature of the matrix lattice^26^. The decay of free radicals can be divided in two phases: the first, corresponding to a “fast” exponential decay, and the second, corresponding to a “slow” linear decay^27^. Because radicals can persist long after radiation exposure ends^22,26–30^, prolonged presence of radical species could contribute to accelerated drug degradation during long-term storage. This contrasts with aqueous solutions where reactive species readily diffuse and can react with drug ingredients to mediate a rapid decrease in drug potency^31^.

The goal of this study is to assess the long-term effect of ionizing radiation on stability of pharmaceuticals by simulating the effect of radiation during spaceflight. This was done by irradiating selected medications using ground-based rapid-switching radiation beam exposures to simulate galactic cosmic radiation (GCR), followed by intermittent testing of drug API and impurity content, and dissolution rate. The drugs used for this study were Acetaminophen, Amoxicillin, Ibuprofen, and Promethazine. These drugs were selected based on previous research experience and relevance to spaceflight. Solid formulations typically have longer shelf lives than non-solid formulations of the same drugs, so that they are more likely to be used for long duration missions. Therefore, only solid dosage forms (tablets or capsules) were used in this study.

## Results

### Environmental conditions

The temperature and relative humidity (RH) of all samples remained within acceptable range for controlled room temperature, as defined by the USP < 659 > ^32^ throughout transport (JSC to and from NSRL; JSC to UMB) and while in storage at JSC and UMB. One brief transient temperature excursion above 30 °C was documented when medications were transported onsite at JSC; however, control and treated samples had the same exposure, and mean kinetic temperature remained within the required range^33^. The summary temperature measurements are provided in Supplemental Methods.

### Irradiation measurements

The thermoluminescence dosimeters (TLDs) with the traveling control group measured radiation exposure of only 1.73 ± 0.15 μGy/day over the course of the experiment, including transit from JSC to NSRL and NRSL to the University of Baltimore Maryland (UMB). The uncertainty is obtained as the standard error from 10 travel TLDs. This shows that background radiation exposure during transport was insignificant relative to the treatment dose.

The detailed TLD-100 passive dosimeter measurements taken during beam exposure are presented in Supplemental Table 3. The entrance dose (“front”) for irradiated drugs at the 500 mGy dose ranged from the lowest measured value of 422.7 ± 5.7 mGy to 465.3 ± 6.3 mGy for ibuprofen and acetaminophen, respectively, resulting in a measured dose of 7–15% lower than the expected nominal dose. The entrance dose for irradiated drugs at the 1000 mGy dose showed similar variations from 864.4 ± 11.7 mGy to 932.4 ± 12.7 mGy for amoxicillin and acetaminophen, respectively, resulting in a measured dose of 7–14% lower than the expected nominal dose. A dose-decreasing trend between the front and back TLDs of 7–16% was observed for each drug group, indicating some degree of radiation absorption. Thus, the average radiation dose was somewhat less than the target dose. This lower-than-expected dose could be due to several factors, notably some shielding in the experimental setting and differences in the methods for estimating dosimetry that have been used.

### API content analysis

**Fig. 1:**
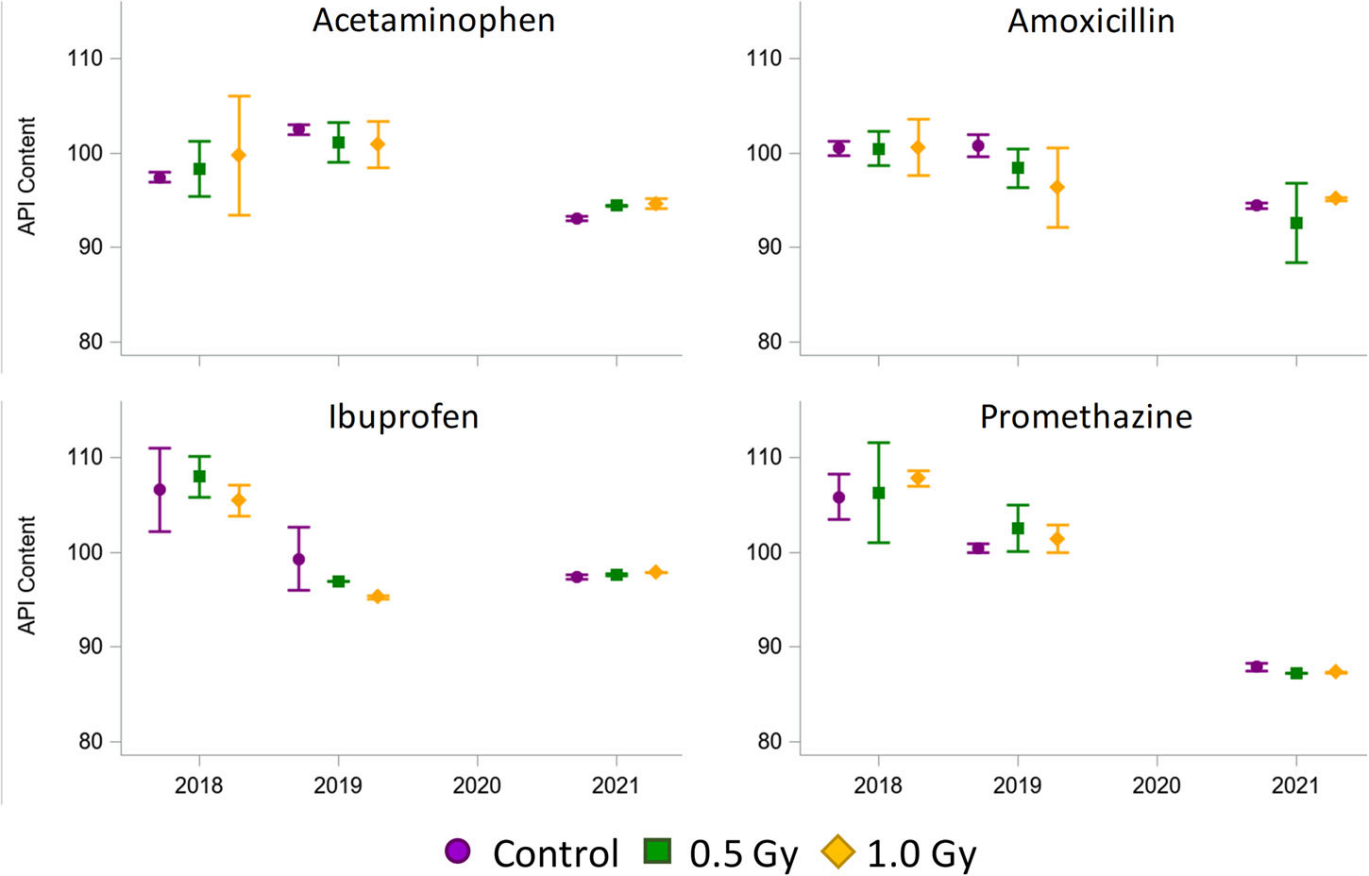
API content. Percent label API content vs time for each drug product as estimated marginal mean ± 95% confidence limits.

The API content of all irradiated medications and matching controls met USP acceptance criteria for potency (90–110%) as shown in Fig. 1. Although the experiment was designed with only two independent replicates (*n* = 2), the variability of the results can be estimated using a mixed effects statistical model.

Across all drugs, API content significantly (*p* < 0.05) decreased over time relative to the first time point (Table 1); however, post-hoc pairwise analysis indicates there is no consistent radiation-dependent effect. There is a large fluctuation in variance across samples, which does not appear to be dependent on treatment or time. These results suggest that ionizing radiation, in the range of the cumulative doses expected for an exploration Mars mission, has no obvious effect on either short-term or long-term drug stability. Estimated marginal means and standard errors from each drug-specific mixed-model, as well as pairwise comparisons, are fully reported in the Supplemental Materials.

**Table 1:** Mixed model tests of fixed effects (condition * year) indicating at least one difference

Effect	Num DF	Den DF	*F* Value	Pr > *F*
Acetaminophen	1	8	36.42	0.0003
Amoxicillin	1	8	445.01	<.0001
Ibuprofen	1	8	20.40	0.0020
Promethazine	1	8	323.16	<.0001

*Num DF* the number of degrees of freedom in the model, *Den DF* the number of degrees of freedom associated with the model errors; *F Value* The F statistic for the given predictor and test statistic, *Pr* *>* *F* the *p*-value associated with the F statistic.

For some drugs, especially acetaminophen, there appears to be a modest increase in API content from earlier time points to later time points; however, this does not appear to be treatment dependent. These changes, in combination with the minimal sample size, suggest that the experimental variance of these results is likely underestimated, even in controls, since the API content of a drug cannot increase with time, so the variability represents measurement error. Consequently, the likelihood of a type I error (“false positive”) is increased, meaning there is a risk that the null hypothesis is erroneously rejected, when in fact there is no difference between treatments. Consistent with this risk, the mixed model ANOVA tests indicate that there is at least one significant difference across Condition and Time combinations; however, the statistical test does not indicate the direction of differences. The pairwise comparisons (Supplemental Table 4) show where differences may be seen with regard to treatment-control. This is important because the API content of some irradiated drugs is higher than the API content of matched controls, whereas the relationship is reversed for other time points. For all these reasons, statistical significance of specific pairwise comparisons should be regarded with some skepticism.

### Impurities

Impurity content over time was assessed for acetaminophen, ibuprofen, and amoxicillin. Promethazine impurities could not be analyzed over the full course of the experiment because an analytical error occurred during the final time point.

**Fig. 2:**
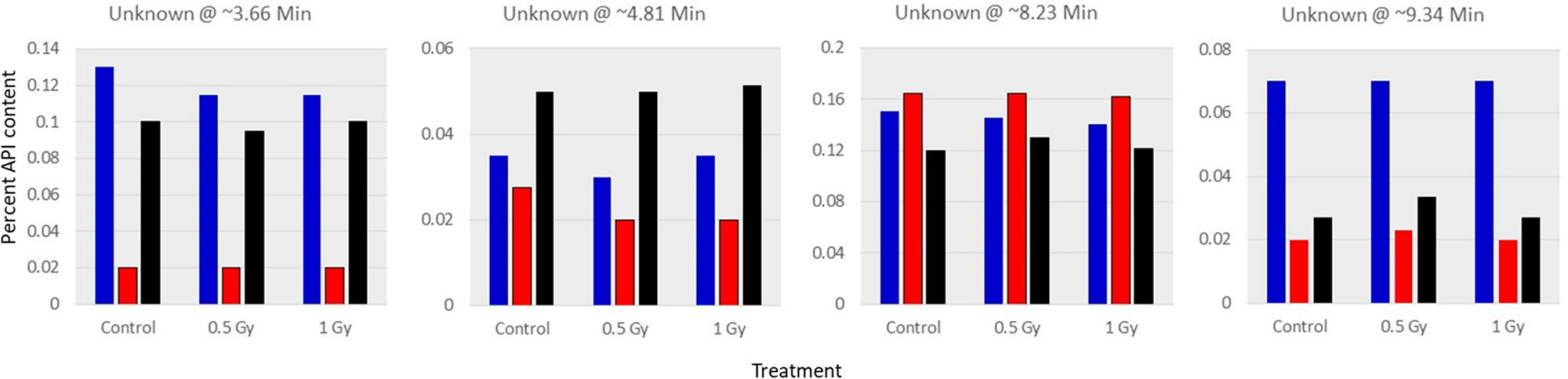
Acetaminophen impurities. Four impurities observed in acetaminophen tablets, identified by chromatographic retention time with levels greater than 0.05%. Blue is 2 months (2018); red is 18 months (2019); black is 34 months (2021). Impurity content is expressed as percent of API in the standard solution.

Acetaminophen tablets do not exhibit a clear radiation-dependent change in impurity content (Fig. 2). The only impurity identified by the USP for acetaminophen tablets is 4-Aminophenol, which was not detected in any sample tested. Several impurities were constantly observed in the control samples over time. All samples met the USP limits of not more than 0.6% for total impurity. However, the 18-Month samples slightly exceeded the content limit for individual unspecified impurities of 0.15%, with all samples having levels of approximately 0.16% for the unknown peak at 8.23 mis. Therefore, based on total impurity content, all samples across all time points met USP specifications for impurities.

**Fig. 3:**
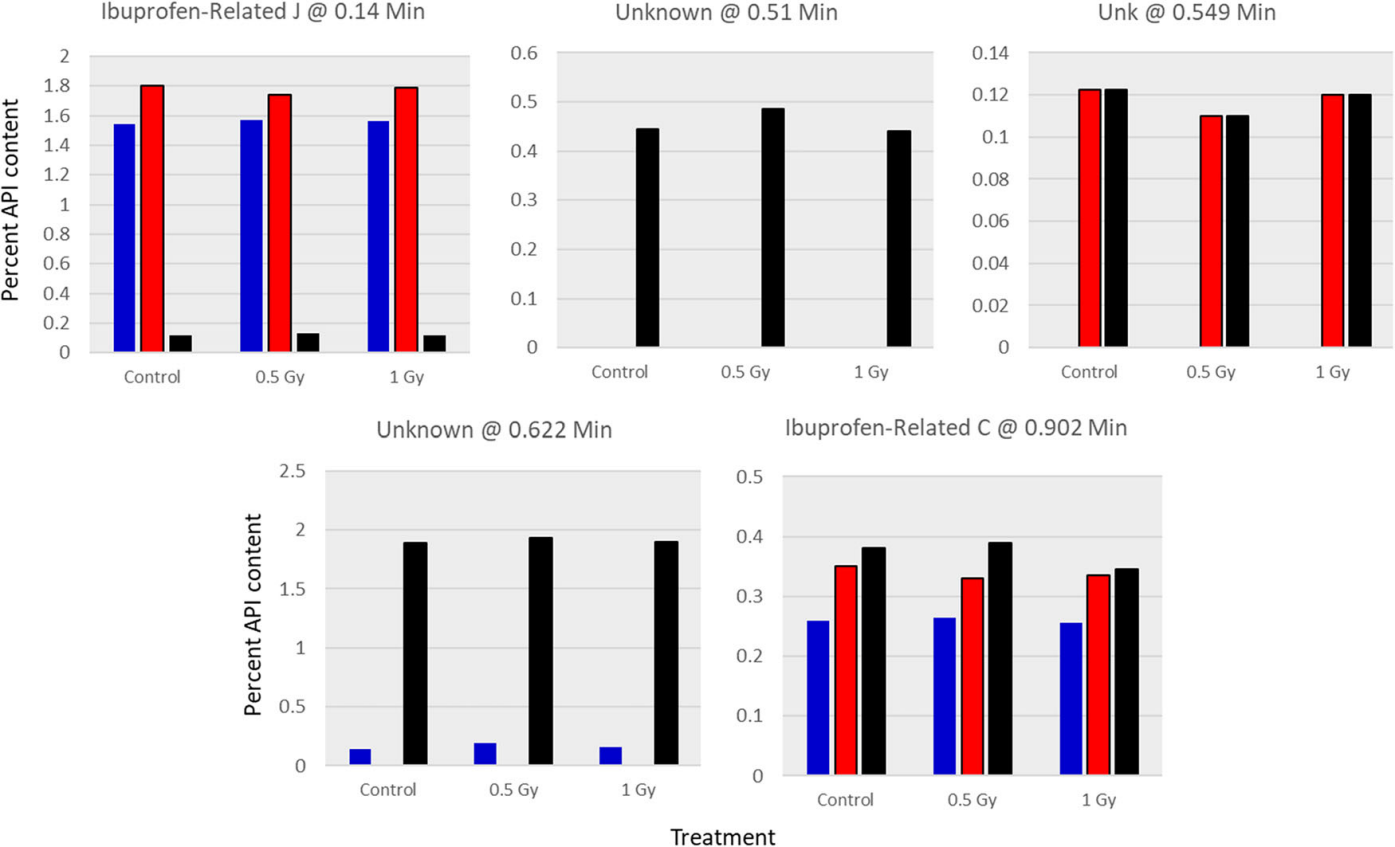
Ibuprofen impurities. Five impurities observed in ibuprofen tablets identified by chromatographic retention time and identity. Blue is 2 months (2018); red is 18 months (2019); black is 34 months (2021). Impurity content is expressed as percent of API in the standard solution.

The UPS acceptance criteria for ibuprofen lists two degradation products: Ibuprofen related compound J and C, with maximum API content limits of 0.2% and 0.25%, respectively. Both impurities are consistently observed across all three time points and treatments (Fig. 3). Ibuprofen related compound J is the only impurity that decreases over time, suggesting it undergoes further degradation to form secondary (daughter) degradation products. Mean levels of compound J exceed USP acceptance limits for all treatments at the first two time points. One unspecified degradation product is observed only at the last time point (Unknown @ 0.51 min), while two other impurities (Unknowns @ 0.549 and 0.622 min)—in addition to ibuprofen related compound C—increase over time. Mean levels of compound C exceed USP acceptance levels at all time points and for all treatments. No radiation-dependent relationship was observed for any impurity since levels were consistent across treatments.

**Fig. 4:**
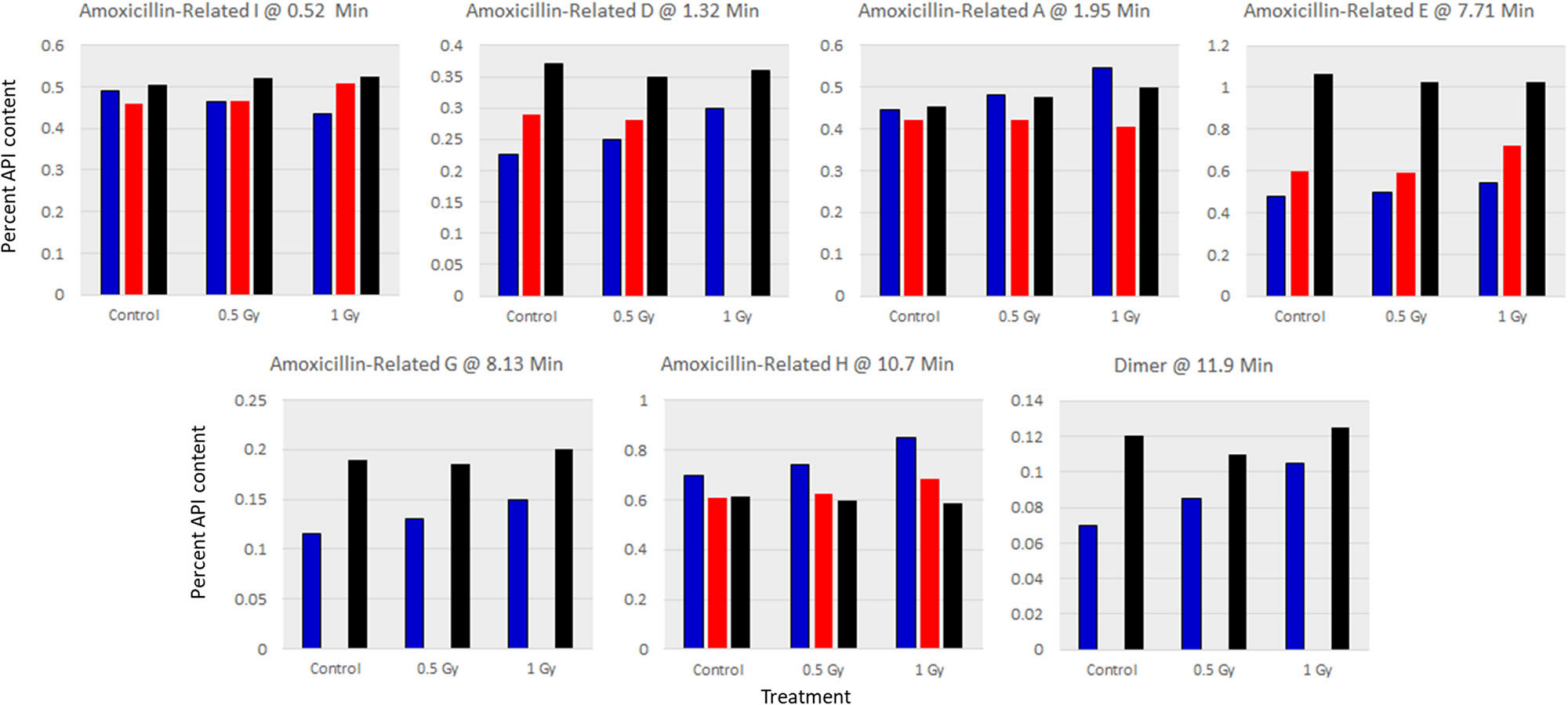
Specified impurities observed in amoxicillin capsules identified by retention time and identity. Blue is 2 months (2018); red is 18 months (2019); black is 34 months (2021). Impurity content is expressed as percent of API in the standard solution.

The USP monograph for Amoxicillin capsules does not specify limits for impurities; however, USP has a general monograph for amoxicillin without regards to dosage form (e.g., capsule, suspension) that does specify impurity limits. Eleven amoxicillin-related impurities are recognized by the general USP amoxicillin acceptance criteria. Seven of these amoxicillin-related impurities were observed in samples from at least two time points (Fig. 4). Two of the impurities show time-related increase across all samples: amoxicillin-related compound D and E. In addition, amoxicillin-related compound G and the amoxicillin dimer appear to increase levels at the last time point relative to the first time point, although no corresponding impurity was observed at the intermediate time point. Irradiation had no clear effect on the content of any of these impurities, although a small increasing trend may be present for some of the identified impurities (e.g., amoxicillin-related compound D) at the first time point (blue), but this change is small, and no impurity exceeded the USP 1% acceptance criteria for individual known impurities. Based on total impurity content, samples were close to the acceptance threshold at the first time point but well below the thresholds at the subsequent 18-month and 34-month time points.

**Fig. 5:**
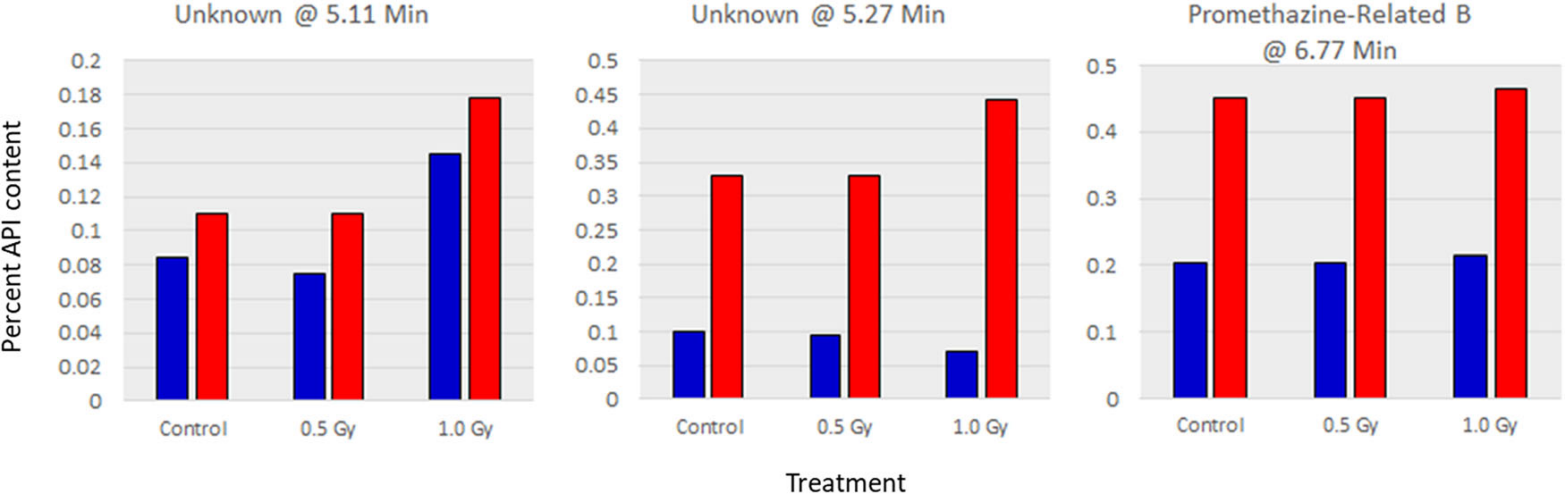
Impurities observed in promethazine tablets identified by retention time and identity. Blue is 2 months (2018); red is 18 months (2019); No results from 34 months are available (2021). Impurity content is expressed as percent of API in the standard solution.

Due to a laboratory error, impurity data for promethazine tablets are available for only the first two time points, at 2 months (2018) and 18 months (2019). There is no apparent effect of radiation treatment on any impurity level at either time point (Fig. 5). The USP acceptance criteria for total degradation products is not more than 1% of API content. This limit excludes promethazine-related compound B, which is a process impurity included in USP specification for identification only and is unchanged by treatment in our samples. Based on total degradation products, at the first time point, all samples met USP impurity acceptance criteria; however, all control and irradiated samples at the 18-month time point fail the impurity quality assessment because they exceeded the USP limit (0.2%) for unspecified degradation products.

Overall, there is no apparent difference in the amount or types of impurities detected in the irradiated samples relative to unirradiated control samples for all drugs tested.

### Dissolution

**Fig. 6:**
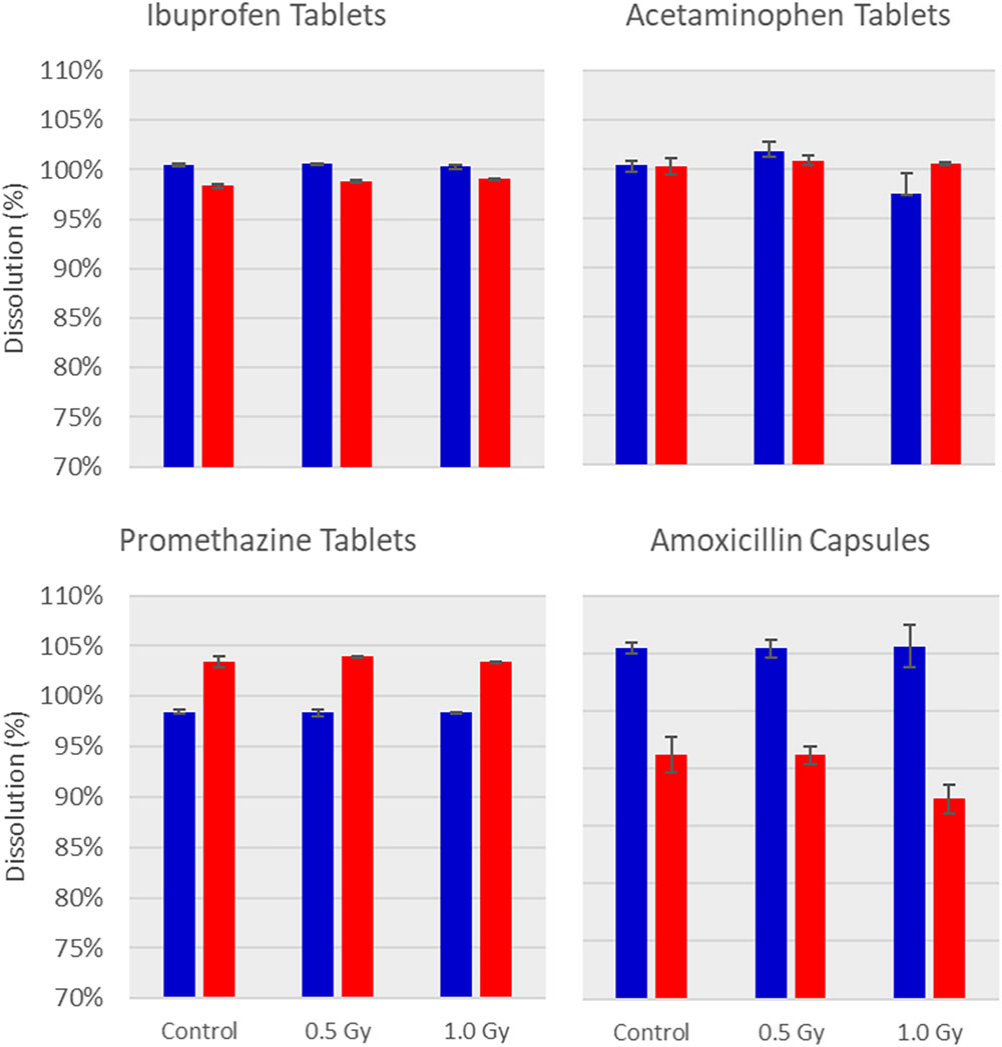
Dissolution experiment results (*n* = 2). Blue is 2-month (2018) samples; red is 18-month (2019) samples. Whiskers indicate the range of data for independent tests on 6 samples.

Dissolution results are shown in Fig. 6. Unfortunately, due to the limited number of available dosing units (i.e., pills) for all drugs at the final time point, dissolution analysis was only performed at 2- and 18-Month (2018 and 2019) time points. All samples met USP requirements for dissolution. Although there are clear time-related changes in dissolution for promethazine and amoxicillin, irradiation exposure does not appear to have a significant effect on dissolution.

## Discussion

API degradation in the solid state is well described^34–38^. Mechanisms typically involve nucleation of degradation reactions (induction) at imperfections in the drug crystal within the dosing unit. The rate of degradation (i.e., the formation of reaction products) is proportional to the number of nuclei formed and proceeds through the growth of each nucleus. The small nuclei in ramified (i.e., imperfect) crystals are unstable and degrade significantly faster than in more perfect crystals. Ionizing radiation can induce damage by exciting molecules, both directly and through radical-cation neutralization reactions^22,30^. Degradation reactions are characterized by a sigmoidal-shaped curve consisting of a lag phase, an acceleration phase, and a deceleration phase. In thermal and photochemical decomposition models, the slow growth (lag) phase is an exponential increase of fractional decomposition with time, over a limited range, as described by the Prout-Tompkins model^34,37,39^. Ionizing radiation excites molecules and forms radicals along tracks, producing high localized concentration of reactive intermediates. Through this process, ionizing radiation can facilitate the initiation of drug degradation reactions, and the presence of excited molecules likely facilitate nuclei expansion. If nucleated sites grow, then this lag phase is followed by an acceleration phase that follows zero-, first or higher order reactions, depending on the drug and experimental conditions^34,35,37^. Based on this mechanistic rationale, it was hypothesized that low-dose ionizing radiation (relative to absorbed doses used for radiosterilization) could initiate the formation of nuclei that are undetectable immediately after irradiation but result in significant degradation over time. In this work, the hypothesis of radiation-induced increased degradation was tested at radiation doses of 0.5 and 1 Gy.

Over the course of this experiment, all four medications exhibit time-dependent loss of API content, with API remaining within the USP-specified range (Table 2). Across all drugs, exposure to ionizing radiation at doses in the range of those expected for long-duration Mars missions do not show any significant effect on the quality of solid-state drugs. Rather, the effect of time was, by far, the greater factor influencing drug stability. The absence of any clear effect on drug stability is likely influenced by both drug-related factors and experimental design.

**Table 2:** Experimental drug list used for this study

Drug	Labeler	Quantity	Packaging	Expiration date^a^	NDC	USP Acceptance Range
Acetaminophen (paracetamol) 500 mg tablets	Major	8 packages (100 units/Pkg)	Manufacture blister packs + Ziplock bags	01/31/2021 05/24/2019	00904-1988-60	90–110%
Amoxicillin 500 mg capsules	Sandoz	8 packages (100 units/Pkg)	Manufacture packaging	12/31/2019	00781-2613-01	90–120%
Ibuprofen 400 mg tablets	Amneal	8 packages (100 units/Pkg)	Manufacture blister packs + Ziplock bags	11/30/2019 05/28/2019	65162-0464-10	90–110%
Promethazine 25 mg tablets	Amneal	8 packages (50 units/Pkg)	Manufacture packaging	02/29/2020	65162-0521-10	95–110%

^a^Manufacturer expiration date.

The primary drug-related factor that contributes to the outcome of this study is the fact that solid-state medications were studied. Exposure to ionizing radiation produces excited and ionized atoms within the drug product. The solid-state matrix prevents diffusion of larger reactive intermediates, essentially trapping them in a “cage”. This cage effect favors geminate recombination of initial radicals before they can diffuse elsewhere and propagate further degradation^22,40,41^. Smaller species, such as electron and hydrogen atoms can diffuse but not as freely as they can in solution. In comparison, aqueous drug solutions are far more sensitive to ionizing radiation at absorbed doses orders of magnitude lower than solid state drugs, which is in part because reactive species can diffuse more freely from their site of formation to mediating degradation chemistry.

Experimental factors contributing to the outcomes of this experiment include the low dose of ionizing radiation to which the medications were exposed (relative to radiation doses used by the pharmaceutical industry radiosterilization). Ionizing radiation in the range of 0.5 to 1 Gy induces the formation of a relatively small number of reactive intermediates, relative to overall molar content of drug in the medications. An analysis by Kim and Plante^42^ modeled the potential effects of radiation on food and pharmaceuticals stored during a 3-year spaceflight journey outside the protection of the Geomagnetosphere. They calculated the mean number of charged particle hits and the radiolytic yields in the target materials of freeze-dried food, intermediate moisture food, and liquid formulation pharmaceuticals. For this assessment, the exterior GCR environment at deep solar minimum was assumed uniform, isotropic, and constant throughout the entire round-trip journey to Mars. It was predicted that GCR is unlikely to cause a rapid change of functional properties in pharmaceuticals stored inside the vehicle, suggesting instead that progressive functional defects would occur with radiation exposure over time. These functional defects would depend on energy deposition, yields of radiolytic species, bond-dissociation frequency, or any other chemical bond breaking phenomena. Kim and Plante also noted that the probability of space radiation hitting individual drug molecules, comprising said drugs—which are predominantly composed of inactive excipients—is very low over a 3-year mission to Mars. On a molar basis, the number of reactive intermediates produced by spaceflight radiation is minuscule relative to the molar concentration of API in most drugs; therefore, the molar change in drug content is clinically inconsequential. Furthermore, radiolytic species, especially the hydroxyl radicals, may not be generated in solid dosage forms due to the low water content of most solid drug products. Therefore, it was concluded that space radiation is not a concern for long-term storage of medications during spaceflight.

It is reasonable to make the assumption that the exposure of drug products to ionizing space radiation will adversely affect stability because many pharmaceutical studies have shown that ionizing radiation causes physical and chemical degradation of drugs^11,12,14–17,43–45^. However, the irradiation used in most of these investigations has important qualitative and quantitative differences from the radiological environment of spaceflight, including the types of radiation, cumulative dose, dose-rate, and the energy spectrum. The most important difference is radiation dose. A Mars transit mission is currently expected to expose astronauts to a cumulative radiation dose in the range of about 1 Gy. Most pharmaceutical radiostability studies are performed in the range of 10 to 100 kGy, which is many orders of magnitude larger than the dose for a Mars mission.

The formation of toxic drug impurities has been proposed to be an important risk for medications during spaceflight, and radiation exposure has been proposed to be a risk factor mediating API degradation and toxicant production. In this study, drug component chromatograms revealed no new or foreign peaks in any of the irradiated drug product samples tested. Moreover, none of the tested drugs exhibited an appearance of radiation-associated impurities. In contrast, several impurities increased with time, regardless of irradiation conditions, consistent with general trend for time-dependent loss of API. It has been previously shown that ionizing radiation does not initiate new degradation pathways; rather, it facilitates reactions that mediate normal drug degradation^14–16,46,47^ In this regard, radiation introduces energy, not new chemical reactions^48^.

The fact that drugs exposed to spaceflight do not form new degradation products when compared to similar terrestrial controls has been previously reported by several NASA-supported studies^19,20,49,50^. Furthermore, there is scientific consensus that radiolytic degradation impurities generated in food substances, at irradiation at doses of up to 10 kGy, pose no toxicological hazard to consumers in the concentrations at which they are detected (Joint FAO/IAEA/WHO Expert Committee 1981). There is no indication that this inference from irradiated food studies would not also apply to pharmaceuticals^16^. A study done using radiation conditions similar to our study^8^ has shown that no nutritional differences were found between the irradiated samples and controls immediately after radiation treatment or 1-year after radiation treatment. Taken together, all available evidence supports a conclusion that prolonged exposure to a spaceflight environment could facilitate degradation of some highly susceptible drugs; however, degradation pathways and reaction products will not be different from those observed in FDA-compliant stability studies. Such studies include stress testing under extreme conditions, accelerated stability testing at elevated temperature and relative humidity, and long-term stability testing^51,52^. When degradation impurities are observed at sufficiently high levels, they must be qualified with regard to their human health risks^53–55^. It is therefore extremely unlikely that environmental conditions associated with human spaceflight will mediate the formation of unexpected, highly potent toxicants that are not already known to form terrestrially, unless medications are exposed to chemical factors capable of mediating unforeseen chemistry. The repackaging of medications into unprotected packaging is one factor that exposes drugs to chemical factors, such as atmospheric gases or vapors present within the spacecraft over a prolonged period.

Du et al.^9^, evaluated the spaceflight stability of three of the drugs used in this study (amoxicillin, ibuprofen, and promethazine) and reported that the spaceflight samples had a greater loss of API relative to lot-matched terrestrial controls^9^. The authors speculated that elevated radiation levels associated with spaceflight may have contributed to increased drug degradation. These results differ from our current findings that equivalent medications exposed to radiation levels approximately ten-fold higher than the maximum absorbed radiation dose reported by Du et al.^9^, have no effect on drug content, impurity levels, or dissolution. In fact, our results are more consistent with other spaceflight drug stability studies that have not observed any unusual changes in drug stability^19,20,49^, including a reanalysis of three drugs originally analyzed by Du et al.^9^. It is more likely that the changes in API content reported by Du et al., are related to repackaging of solid oral medications (capsules and tablets) into non-protective containers combined with prolonged storage, which resulted in the quality failure of many medications in both the terrestrial and spaceflight treatment groups prior to their labeled expiration dates^21^.

Dissolution releases API from solid-state pharmaceuticals, and altered dissolution can reflect changes in the composition of tablets or capsules. There are many changes that can account for altered dissolution, including increased polymerization of tablet coating polymers, altered API crystal morphology, and altered moisture content, among others^56,57^. Over the course of the experiment, dissolution decreased for amoxicillin, resulting in a slightly slower release of API release^58^, and slightly faster for promethazine, resulting in a slightly more rapid release of API. No substantive temporal change occurred^9^ with acetaminophen or ibuprofen. Importantly, across all time points, no functionally important difference in the dissolution was associated with ionizing radiation exposure.

There are several substantive limitations of this study. First and foremost is that only two independent replicates were tested for each treatment condition. Two replicates are inadequate for determining variability and provide insufficient statistical power needed to test the hypothesis that irradiation affects drug stability. One of these choices included heavy emphasis on USP methods which emphasize the use of pooled dosing units of not less than 10 or 20 dosing units. This choice reduces sampling variability at the expense of statistical variance information. To overcome this issue, we used a mixed model ANOVA analysis to estimate the mean and standard error of API content results. This analysis supports the null hypothesis that API content is similar between the irradiated and control samples with no substantial degradations in API compared to the JSC experimental controls. However, the risk of committing a type II error (falsely failing to reject the null hypothesis that there is no difference between treatments) is high because of the small number of replicates. Therefore, given the minimal sample tested for this study, results should be considered exploratory and need to be confirmed in future studies. This study provides the necessary initial estimates to conduct power assessments for planning future studies.

Another limitation of this study relates to the testing and analysis workflow. The study started in 2018 and lasted for over three years. During that time, operations have been disrupted in part due to the coronavirus pandemic, and personnel have changed. Unfortunately, for these reasons, several experimental results are missing. Although not critical, the environmental monitoring data for the transportation between JSC and UMB is missing. The detailed chromatographic reports for API assays and impurities are missing entirely for 2018; fortunately, the summary reports are available. The dissolution assays were done in 2018 and 2019 only. Obviously, it would be useful to repeat the experiment to capture the missing data points, which would help strengthen the conclusions of this study. However, this experiment lasted for three years and involved coordination between several institutions. Also, it would be surprising if the inclusion of the missing data points changed the conclusions.

This study has shown that some degradation of the drugs occurs during the 3-year period, as evidenced by a decrease in the API, change in the impurity content, and some change in the dissolution. Furthermore, no significant differences in the results were observed between the irradiated and controls groups, which was the primary focus of this work. This is not surprising, as the radiation doses used (1 Gy or less) are several orders of magnitude lower than those commonly used for sterilization of solid-state drugs. It is likely that drug re-packaging, environmental conditions, and time have a greater influence on degradation than ionizing radiation in a range that is acceptable for human exposure.

Accurately characterizing the effects of GCR is critical to the success of long duration spaceflight missions. The results obtained from this simulated GCR exposure drug stability study are one step in characterizing the effects of the space environment on medication stability. These data—along with that obtained from additional studies—can be used to generate estimates for medication shelf life and time-to-failure intervals for flight medications and identify the best candidates for an exploration spaceflight medication formulary. For future experiments on the radiostability of drugs, it would be useful to use non-solid formulation, especially since they would have some water content. That is, irradiation of non-solid formulations would lead to the formation of water radiolysis products, such as the hydroxyl radical, which could cause degradation of the medication. It could also be useful to use higher radiation doses to characterize the shape of the dose-effect curve. This will give a measure of the margin of safety (MoS) for drug radiation exposure. For example, if degradation is seen at 20 Gy but not at 10 Gy, then this would indicate that there is a MoS of at least 10-fold for a cumulative dose expected over a Mars mission ( ~ 1 Gy). As space exploration moves beyond low Earth orbit (LEO), it is critical that NASA continues its commitment to crew health and safety by continuing to track the space environment effect on medication performance and seeking mitigation strategies to combat therapeutic failure.

## Methods

### Pharmaceutical products and reagents

Four commercially available medications were procured by the NASA pharmacy. Each drug product was from identical manufacturing lots for each treatment arm. Thirty-two (32) packages of medications (2 packages for each of the 4 selected medications and for each of the 4 study conditions) were purchased. The medications are described in Table 2.

According to the FDA, the drug expiration dates reflect the period during which the product is known to remain stable, which means it retains its strength, quality, and purity when it is stored according to its labeled storage conditions. For most drugs, USP specifications require at least 90% of the original potency at the expiration date. Technically, drugs should not be used past their labeled expiration date. However, for most drugs, the potency decreases gradually over time and continues to meet USP specifications well past their expiration date. For most medicines, a small reduction in potency is not clinically important. Furthermore, most drugs are not harmful after they expire. Whether a drug can be used past the expiration date is debated and depends on the type of drug and the clinical indication. The typical expiration date for prescription products, i.e., shelf-life, is usually 12–60 months. For a mission such as the Mars Mission, which may have a duration of about three years, it is likely that some drugs will be beyond their expiration dates at some point during the mission.

### Treatment groups

Drugs were assigned to three treatment groups:

1. The non-irradiated experimental control group. These samples had the same environmental exposures as the treatment samples, including transport and storage.
2. The irradiation group I (GCRSim, up to 0.5 Gy total requested dose).
3. The irradiation group II (GCRSim, up to 1.0 Gy total requested dose).

A group of matched, non-irradiated medications underwent the exact same environmental exposures as the radiation-treated groups, aside from irradiation exposure, including transportation between study sites and storage. This group serves as the reference group for all analyses. A separate control group was retained only at Johnson Space Center (JSC) throughout the duration of the experiment to assure that the multiple logistical steps didn’t affect drug stability independently of the radiation treatment. This group was the JSC reference samples.

### Packaging

Each drug product was packaged to resemble, as closely as possible, the operational packaging used in flight medical systems (e.g. drug flight bottles/bags/unit-dose strips). Promethazine and amoxicillin tablets were maintained in their sealed manufacturer container throughout the duration of the experiment until analytical testing. Acetaminophen and ibuprofen were removed from the manufacturer’s outer packaging and repackaged into zip-lock bags but kept in manufacturer’s unit dose packages. Repackaged Ibuprofen was further protected by repackaging into sealed amber, light-resistant bags (Supplement Fig. 1).

### Logistics, storage and environmental monitoring

Drugs were shipped from NASA JSC to NSRL at Brookhaven National Laboratory utilizing a contracted courier for overnight delivery. Precautions were taken to assure medications remained within labeled environmental conditions. USP specifies controlled room temperature (CRT) as 20 to 25 °C with allowable excursion between 15 and 30 °C^32,59^. The shipping courier provided temperature tracking using a Sensitech® Temp Tale 4 temperature monitor (Supplement Table 2A). Additional temperature tracking during shipment used a LogTag® TRIX-8 model temperature tracking device (Supplement Table 2B). Silica gel desiccant pouches were included in the shipment package to reduce exposure to RH for repackaged drugs; silica pouches were included in the sealed, manufacturer packaging. Additionally, passive thermoluminescence dosimeters (TLD-100 LiF:Mg,Ti)^60^, provided by NASA-JSC Space Radiation Analysis Group (SRAG), were included with medications during shipment.

Treatment group medications were irradiated together on study day Zero, then—along with the untreated controls—returned to JSC by courier with similar environmental monitoring precautions. Upon the receipt of medications at JSC, the NSRL samples joined the JSC control group medications in environmentally controlled storage chambers (Darwin Chambers KB0303), where they remained for 47 days, until shipped to the University of Maryland, Baltimore (UMB) Applied Pharmaceutics Lab for long-term storage and quality testing. A full set of samples was removed from storage and underwent quality testing at 2 months, 18 months, and 34 months after irradiation (Supplementary Table 1).

JSC and UMB storage conditions were similar. Medications were stored under dry, room temperature conditions at approximately (20 ^o^C/30% relative humidity). UMB utilized a Caron Environmental Chamber Model 7000-10 (CARON Products & Services, Inc. Marietta, OH, USA).

### Radiation exposure and monitoring

**Fig. 7:**
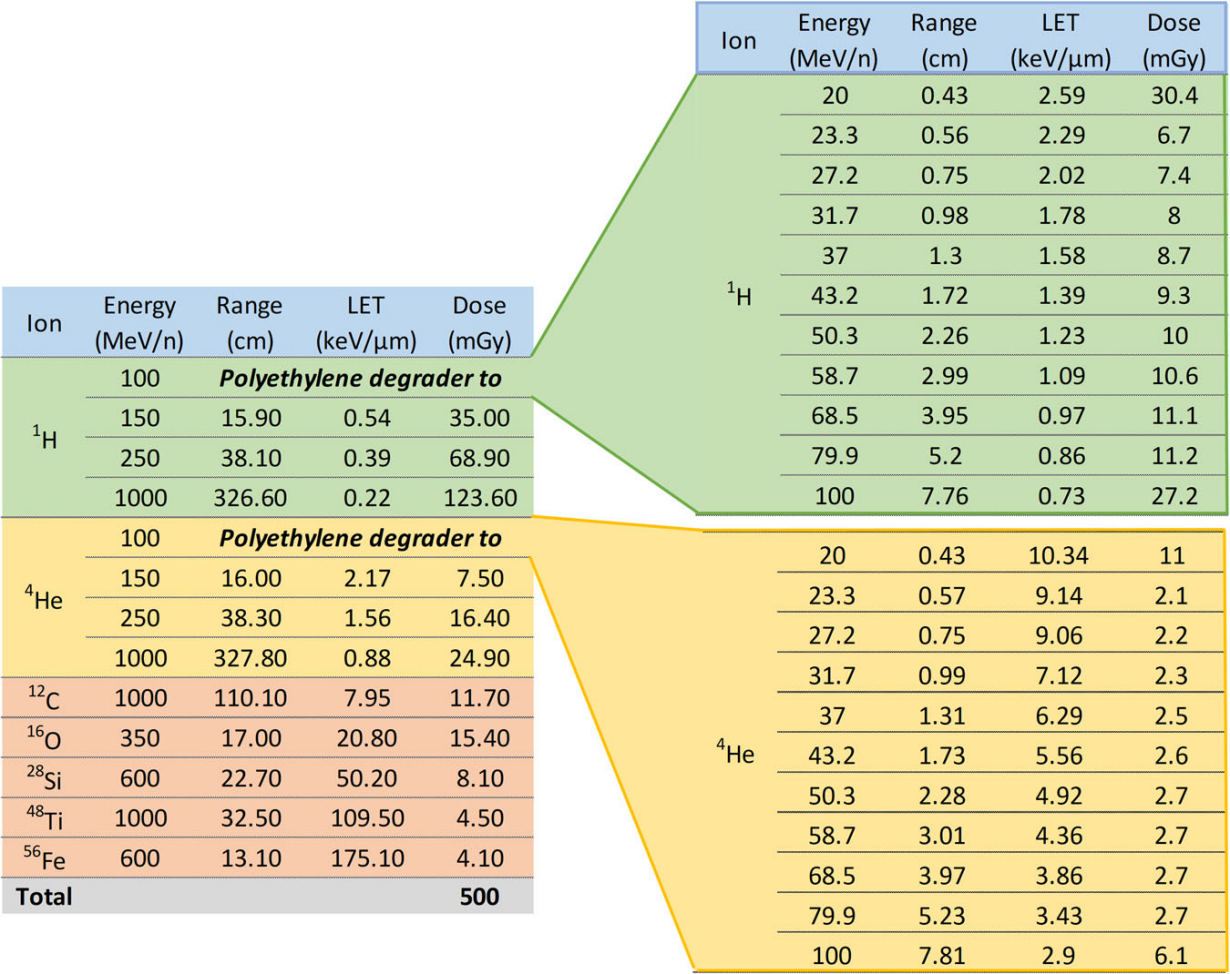
NSRL GCR Simulation Beam Composition as characterized at https://www.bnl.gov/nsrl/userguide/gcrsim.php.

Drugs were exposed to a GCR-like beam profile consisting of ^1^H, ^4^He, ^12^C, ^16^O, ^28^Si, ^48^Ti, and ^56^Fe ions^61^. The target radiation doses for the mixed beams were 0.5 Gy and 1.0 Gy. The detailed NSRL GCR simulation (GCRSim) beam composition profile^62^ used for this experiment is shown in Fig. 7 for the 0.5 Gy dose. The “low-LET” (<5 keV/μm) dose contribution to the total GCRSim dose was 86.7%, while the “high-LET” (>5 keV/μm) dose contribution to the total dose was 13.3%. Target radiation dose was verified using a set of 32 Thermoluminescence Dosimeters (TLD-100 LiF:Mg,Ti) provided by the NASA-JSC SRAG. Some of the dosimeters were used to measure the background radiation dose during travel. The TLDs were carried by plane in a lead bag from JSC to NSRL and from NSRL back to JSC. The TLDs were placed in clear gelatin capsules (Lilly, No. 0) for irradiation. The TLD capsules were attached to either the back or front and back of the two packages of each drug product (Supplementary Fig. 1). NASA-JSC Biomedical Research and Environmental Sciences Division and SRAG scientists quantified the total dosimeter radiation exposure; results are provided in Supplementary Table 3.

### Drug quality analyses

All API and impurity standards were purchased from USP (Rockville, MD). Solvents used for analysis met or exceeded high performance liquid chromatography (HPLC) grade. Reagents used for processing samples were at minimum American Chemical Society (ACS) grade.

API assay was performed at the UMB Applied Pharmaceutics Lab. Stability analyses consisted of three quality assessments based on USP drug monographs and International Council for Harmonisation (ICH) Q6A guidance: API content analysis (i.e., “assay”), API release characteristics (dissolution), and identification of degradation impurities. UMB analytical protocols for API content (TMD-013, TMD-016, TMD-020, and TMD-022), impurities (TMD-014, TMD-017, TMD-020 and TMD-023), and dissolution (TMD-015, TMD-018, TMD-021 and TMD-024) are given in the Supplementary Methods. A protocol deviation is noted for the analysis of Promethazine 25 mg tablets (TDM-019), which did not adequately separate the promethazine peak from impurities. Method TMD-020, which did provide good separation of peaks, was used for the API assay at the first two time points. However, at the last time point, TDM-019 was mistakenly used for analysis of impurities which prevented reliable alignment of discrete impurity peaks with peaks observed at earlier time points. For this reason, it is not possible to evaluate impurities in the promethazine tabels at the final time point.

USP quality testing methods for promethazine hydrochloride tablets and amoxicillin capsules specify pooling 20 tablets/capsules for extraction and analysis. For acetaminophen and ibuprofen, USP methods specify a composite of not less than 10 tablets. Pooling multiple dose units is recommended for reducing variability associated with individual dosing units^63^. API and impurity content were measured in two independent replicates for each drug product for each treatment condition. API and impurity content were calculated based on peak area relative to known amounts of USP API standards (Supplementary Methods).

Impurities that exceeded 0.05% of the peak chromatographic response of the API standard were analyzed for difference over time. This is a practical threshold for analysis since matching peaks across multiple time points for very low-concentration analytes is challenging and many analytes are variably detected. The UMB monographs containing information on the chromatographic peaks and the detailed results are included in the Supplementary Methods.

Dissolution analysis was performed using a Hanson Vision Elite 8 dissolution apparatus (Teledyne Instruments Hanson Research) in accordance with USP Dissolution method apparatus requirements^64^. Mean and standard deviation were calculated for six experimental replicates. The conditions used for dissolution testing are shown in Table 3. The experiment was performed at 37 ^o^C, as specified in USP^64^.

**Table 3:** Conditions for dissolution testing

Drug	Dissolution time	Buffer condition	Agitation rate (rpms)
Acetaminophen	30 min	pH 5.8 Phosphate Buffer, 900 mL	50 rpm
Amoxicillin	60 min	Water, 900 mL	75 rpm
Ibuprofen	60 min	pH 7.2 Phosphate Buffer, 900 mL	50 rpm
Promethazine	45 min	0.01 N Hydrochloric Acid, 900 mL	100 rpm

### Statistical analysis

Mixed effects models were used to analyze API content defined in terms of the interaction of Condition (JSC control, traveling control, radiation 0.5 Gy dose, radiation 1 Gy dose) and Time (year) as categorical fixed effects. Models contained package-specific GEE (generalized estimating equation) effects to address the repeated measures and robust standard errors to allow non-homogenous variance across Condition and Time. Separate models were fit for each drug. Residuals were inspected to ensure adequate compliance with normality assumptions. Estimated marginal means were used to estimate mean API content for each Condition at each Time. An overall F-test of the interaction of Condition*Time was deemed significant before proceeding with pairwise comparisons which focused on the change from baseline (2018) within each group at later time points, as well as between each group and the control Condition (“traveling control”) at each time point. Analysis was performed in the SAS v9.4 software using the GLIMMIX procedure with the LSMEANS statement. The GLIMMIX procedure fits general linear statistical models without assuming consistent variability normally distributed response. Complete SAS output results are provided in the API Analysis section of the Supplementary Information. Given the limited sample sizes, we consider these analyses exploratory, and chose to not conduct multiple testing adjustments.

Although impurity analyses were repeated in triplicate on each sample, raw data of statistical analysis are only available for about half of the samples (only summary means are available for 2018; some raw data are missing for the 2019 and 2021 time points). Mean impurity content is available for all samples but without corresponding variance (e.g., standard deviation). For this reason, it is not possible to statistically test whether radiation treatment has effects on impurity content. Additionally, data for promethazine impurities are not available due to an analytical error that made the alignment of chromatographic peaks unreliable.

Dissolution results are only available for the first two time points; no results are available for the 34-month (2021) time point, due to an insufficient amount of sample. Since raw data are not available for dissolution studies—and since only two independent samples were analyzed—statistical analysis was not performed.

## Supplementary information

Supplemental material

## Data Availability

The data used in the article can be accessed here: https://figshare.com/articles/dataset/Long-Term_stability_of_irradiated_solid-state_pharmaceuticals/28083524.
