# Supplementary material for: The long-term stability of solid-state oral pharmaceuticals exposed to simulated intravehicular space radiation
Source: NPJ Microgravity. 2025 May 17;11:17. doi: 10.1038/s41526-025-00469-w (PMC12085706; doi:10.1038/s41526-025-00469-w)
Supplement: Supplementary file 1 — Supplemental material [file 41526_2025_469_MOESM1_ESM.pdf]

**Supplementary information**

**Supplementary Table 1**. Key experimental event dates

| | **JSC control** | **Experimental control** | **Irradiation I** | **Irradiation II** |
|---|---|---|---|---|
| **Transport JSC to NSRL** | N/A | 06/06/2018-06/09/2018 | 06/06/2018-06/09/2018 | 06/06/2018-06/09/2018 |
| **Irradiation (Study Day Zero)** | N/A | N/A | 06/12/2018 | 06/12/2018 |
| **Transport NSRL to JSC** | N/A | 06/14/2018-06/18/2018 | 06/14/2018-06/18/2018 | 06/14/2018-06/18/2018 |
| **Transport JSC to UMB** | 07/31/2018-08/02/2018 | 07/31/2018-08/02/2018 | 07/31/2018-08/02/2018 | 07/31/2018-08/02/2018 |
| **First analysis timepoint (2 months)** | 08/2018 | 08/2018 | 08/2018 | 08/2018 |
| **Second analysis timepoint (18 months)** | 11/2019-12/2019 | 11/2019-12/2019 | 11/2019-12/2019 | 11/2019-12/2019 |
| **Third analysis timepoints (34 months)** | 04/2021-05/2021 | 04/2021-05/2021 | 04/2021-05/2021 | 04/2021-05/2021 |

**Summary statistics for temperature tracking**

**Supplemental Table 2A**: Summary statistics for temperature tracking from 06/06/2018 to 06/18/2018, provided by TempTale 4 USB.

| | |
|---|---|
| First timepoint | 06/06/2018, 4:02:18 AM GMT |
| Last timepoint | 06/18/2018, 6:32:40 PM GMT |
| Number of points | 3631 |
| Trip length | 12 days, 14 hours, 35 mins |
| Low alarm limit | 15.0 ˚C |
| High alarm limit | 25.0 ˚C |
| Mean ± std. dev. | 21.2 ˚C ± 1.5 ˚C |
| Mean Kinetic Temperature | 21.4 ˚C |
| Low extreme | 18.9 ˚C |
| High extreme | 25.8 ˚C |
| Time below | 0 sec (0 events) |
| Time above | 1 hr 10 mins (1 event) |

**Supplemental Table 2B**: Summary statistics for temperature tracking from 06/06/2018 to 06/18/2018, provided by Log Tag TRIX-8 temperature sensor.

| | |
|---|---|
| First timepoint | 06/06/2018, 11:59:28 AM UTC |
| Last timepoint | 06/18/2018, 02:59:29 PM UTC |
| Number of points | 3493 |
| Trip length | 12 days, 3 hours, 5 mins |
| Low alarm limit | 2.0 ˚C |

| | |
|---|---|
| High alarm limit | 5.0 ˚C |
| Mean ± std. dev. | 21.6 ˚C ± 1.5 ˚C |
| Mean Kinetic Temperature | 21.7 ˚C |
| Low extreme | 19.1 ˚C |
| High extreme | 31.4 ˚C |

**Supplemental Table 3.** Summary of TLD-100 Dose Measurement Results

| Drug Type | Exposure | TLD-100 Measured Dose (mGy) | TLD-100 Mean Dose (mGy) | TLD-100 Ratio Back/Front | Nominal NSRL Dose (mGy) |
|---|---|---|---|---|---|
| Acetaminophen 500mg | A3a_Front | 465.3 ± 6.3 | 448.1 ± 6.1 | 0.93 ± 0.02 | 500 |
| | A3a_Back | 431.0 ± 5.9 | | | 500 |
| | A3b_Back | 412.7 ± 5.6 | 412.7 ± 8.8 | N/A | 500 |
| Acetaminophen 500mg | A4a_Front | 932.4 ± 12.7 | 899.2 ± 9.8 | 0.93 ± 0.02 | 1000 |
| | A4a_Back | 866.0 ± 11.8 | | | 1000 |
| | A4b_Back | 843.9 ± 11.5 | 843.9 ± 11.5 | N/A | 1000 |
| Amoxicillin 500mg | B3a_Front | 436.2 ± 5.9 | 400.7 ± 5.5 | 0.84 ± 0.02 | 500 |
| | B3a_Back | 365.2 ± 5.0 | | | 500 |
| | B3b_Back | 371.9 ± 5.1 | 371.9 ± 5.1 | N/A | 500 |
| Amoxicillin 500mg | B4a_Front | 864.4 ± 11.7 | 804.4 ± 9.0 | 0.86 ± 0.02 | 1000 |
| | B4a_Back | 744.4 ± 10.1 | | | 1000 |
| | B4b_Back | 747.0 ± 10.2 | 747.0 ± 10.2 | N/A | 1000 |
| Ibuprofen 400mg | C3a_Front | 422.7 ± 5.7 | 405.7 ± 5.5 | 0.92 ± 0.02 | 500 |
| | C3a_Back | 388.8 ± 5.3 | | | 500 |
| | C3b_Back | 394.4 ± 5.4 | 394.4 ± 5.4 | N/A | 500 |
| Ibuprofen 400mg | C4a_Front | 871.5 ± 11.8 | 822.6 ± 9.2 | 0.89 ± 0.02 | 1000 |
| | C4a_Back | 773.7 ± 10.5 | | | 1000 |
| | C4b_Back | 733.3 ± 10.0 | 733.3 ± 10.0 | N/A | 1000 |
| Promethazine 25mg | E3a_Front | 448.4 ± 6.1 | 413.8 ± 5.6 | 0.85 ± 0.02 | 500 |
| | E3a_Back | 379.2 ± 5.2 | | | 500 |
| | E3b_Back | 400.4 ± 5.4 | 400.4 ± 5.4 | N/A | 500 |
| Promethazine 25mg | E4a_Front | 923.6 ± 12.6 | 847.5 ± 9.7 | 0.84 ± 0.02 | 1000 |
| | E4a_Back | 771.5 ± 10.5 | | | 1000 |
| | E4b_Back | 769.4 ± 10.5 | 769.4 ± 10.5 | N/A | 1000 |

[1.] The TLD measured dose values include the control dose subtraction, no additional corrections needed.

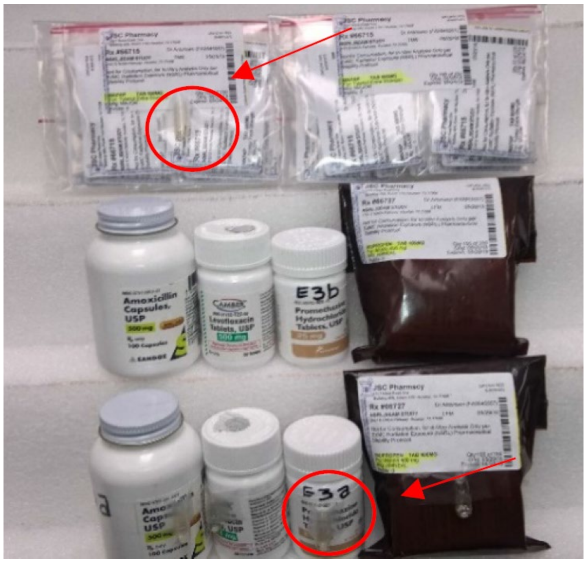


**Supplement Figure 1**. Experimental setup of the irradiation of drugs at NSRL, showing the TLD placement on the samples (red arrows and circles). From Daniels, NASA Technical Report Document ID: 20220000281, Presentation, ExMC GROUND-BASED SPACE RADIATION ANALOG PILOT DRUG STABILITY STUDY: FINAL DATA REVIEW. September 28, 2021.

**Supplemental Table 4**. Pairwise Comparisons for the effect of radiation on each drug

**Simple Effect Comparisons of condition*year Least Squares Means By year**

**Acetaminophen**

| Simple Effect Level | Condition | Condition | Estimate | Standard Error | DF | t Value | Pr > \|t\| | Alpha | Lower | Upper |
|---|---|---|---|---|---|---|---|---|---|---|
| year 2018 | 0.5Gy | traveling control | 0.9400 | 1.5274 | 8 | 0.62 | 0.5554 | 0.05 | -2.5821 | 4.4621 |
| year 2018 | 1Gy | traveling control | 2.3100 | 2.5668 | 8 | 0.90 | 0.3944 | 0.05 | -3.6090 | 8.2290 |
| year 2019 | 0.5Gy | traveling control | -1.3400 | 1.1384 | 8 | -1.18 | 0.2730 | 0.05 | -3.9653 | 1.2853 |
| year 2019 | 1Gy | traveling control | -1.6350 | 1.3117 | 8 | -1.25 | 0.2478 | 0.05 | -4.6597 | 1.3897 |
| year 2021 | 0.5Gy | traveling control | 1.3200 | 0.04950 | 8 | 26.67 | <.0001 | 0.05 | 1.2059 | 1.4341 |
| year 2021 | 1Gy | traveling control | 1.5050 | 0.3288 | 8 | 4.58 | 0.0018 | 0.05 | 0.7468 | 2.2632 |

**Amoxicillin**

| Simple Effect Level | Condition | Condition | Estimate | Standard Error | DF | t Value | Pr > \|t\| | Alpha | Lower | Upper |
|---|---|---|---|---|---|---|---|---|---|---|
| year 2018 | 0.5Gy | traveling control | -0.06000 | 0.4738 | 8 | -0.13 | 0.9023 | 0.05 | -1.1525 | 1.0325 |
| year 2018 | 1Gy | traveling control | 0.08000 | 1.6263 | 8 | 0.05 | 0.9620 | 0.05 | -3.6704 | 3.8304 |
| year 2019 | 0.5Gy | traveling control | -2.3700 | 0.3818 | 8 | -6.21 | 0.0003 | 0.05 | -3.2505 | -1.4895 |

| Simple Effect Comparisons of condition*year Least Squares Means By year | | | | | | | | | | |
|---|---|---|---|---|---|---|---|---|---|---|
| **Acetaminophen** | | | | | | | | | | |
| **Simple Effect Level** | **Condition** | **Condition** | **Estimate** | **Standard Error** | **DF** | **t Value** | **Pr > \|t\|** | **Alpha** | **Lower** | **Upper** |
| year 2019 | 1Gy | traveling control | -4.4200 | 1.3294 | 8 | -3.32 | 0.0105 | 0.05 | -7.4855 | -1.3545 |
| year 2021 | 0.5Gy | traveling control | -1.8300 | 2.0082 | 8 | -0.91 | 0.3888 | 0.05 | -6.4609 | 2.8009 |
| year 2021 | 1Gy | traveling control | 0.6850 | 0.06718 | 8 | 10.20 | <.0001 | 0.05 | 0.5301 | 0.8399 |

| Ibuprofen | | | | | | | | | | |
|---|---|---|---|---|---|---|---|---|---|---|
| **Simple Effect Level** | **condition** | **condition** | **Estimate** | **Standard Error** | **DF** | **t Value** | **Pr > \|t\|** | **Alpha** | **Lower** | **Upper** |
| year 2018 | 0.5Gy | traveling control | 1.3750 | 2.8956 | 8 | 0.47 | 0.6476 | 0.05 | -5.3023 | 8.0523 |
| year 2018 | 1Gy | traveling control | -1.1750 | 2.6623 | 8 | -0.44 | 0.6706 | 0.05 | -7.3142 | 4.9642 |
| year 2019 | 0.5Gy | traveling control | -2.3200 | 1.4779 | 8 | -1.57 | 0.1551 | 0.05 | -5.7279 | 1.0879 |
| year 2019 | 1Gy | traveling control | -4.0300 | 1.3930 | 8 | -2.89 | 0.0201 | 0.05 | -7.2423 | -0.8177 |
| year 2021 | 0.5Gy | traveling control | 0.2200 | 0.1626 | 8 | 1.35 | 0.2131 | 0.05 | -0.1550 | 0.5950 |
| year 2021 | 1Gy | traveling control | 0.4600 | 0.1202 | 8 | 3.83 | 0.0050 | 0.05 | 0.1828 | 0.7372 |

| Promethazine | | | | | | | | | | |
|---|---|---|---|---|---|---|---|---|---|---|
| **Simple Effect Level** | **condition** | **condition** | **Estimate** | **Standard Error** | **DF** | **t Value** | **Pr > \|t\|** | **Alpha** | **Lower** | **Upper** |
| **year 2018** | **0.5Gy** | **traveling control** | 0.4400 | 3.3658 | 8 | 0.13 | 0.8992 | 0.05 | -7.3216 | 8.2016 |
| **year 2018** | **1Gy** | **traveling control** | 1.9900 | 0.6930 | 8 | 2.87 | 0.0208 | 0.05 | 0.3920 | 3.5880 |
| **year 2019** | **0.5Gy** | **traveling control** | 2.1250 | 1.2763 | 8 | 1.66 | 0.1345 | 0.05 | -0.8182 | 5.0682 |
| **year 2019** | **1Gy** | **traveling control** | 1.0300 | 0.8344 | 8 | 1.23 | 0.2521 | 0.05 | -0.8941 | 2.9541 |
| **year 2021** | **0.5Gy** | **traveling control** | -0.6550 | 0.1732 | 8 | -3.78 | 0.0054 | 0.05 | -1.0545 | -0.2555 |
| **year 2021** | **1Gy** | **traveling control** | -0.5700 | 0.1344 | 8 | -4.24 | 0.0028 | 0.05 | -0.8798 | -0.2602 |

## Supplemental Table 5. API content results

| | | | Treatment Condition | | | | | | | |
|---|---|---|---|---|---|---|---|---|---|---|
| | | Approx analysis dates | JSC Control A | JSC Control B | Exper. Control A | Exper. Control B | 0.5 Gy A | 0.5Gy B | 1 Gy A | 1 Gy B |
| | | | A1A | A1B | A2A | A2B | A3A | A3B | A4A | A4B |
| Acetaminophen | 2018 | 8/1/2018 | 95.3 | 100.4 | 97.08 | 97.73 | 100.18 | 96.51 | 95.76 | 103.67 |
| | 2019 | 11/1/2019 to 12/31/19 | 103.22 | 101.85 | 102.18 | 102.81 | 102.45 | 99.86 | 102.4 | 99.32 |
| | 2021 | 4/1/2021 to 5/31/21 | 94.72 | 96.17 | 92.97 | 93.24 | 94.36 | 94.49 | 94.94 | 94.28 |
| | | | C1A | C1B | C2A | C2B | C3A | C3B | C4A | C4B |
| Ibuprofen | 2018 | 8/1/2018 | 103.85 | 106.6 | 109.32 | 103.84 | 106.6 | 109.31 | 104.38 | 106.43 |
| | 2019 | 11/1/2019 to 12/31/19 | 98.24 | 102.94 | 97.21 | 101.37 | 96.98 | 96.96 | 95.15 | 95.37 |
| | 2021 | 4/1/2021 to 5/31/21 | 97.01 | 96.81 | 97.22 | 97.55 | 97.67 | 97.54 | 97.85 | 97.84 |
| | | | E1A | E1B | E2A | E2B | E3A | E3B | E4A | E4B |
| Promethazine | 2018 | 8/1/2018 | 99.17 | 104.66 | 107.32 | 104.33 | 103 | 109.53 | 108.33 | 107.3 |
| | 2019 | 11/1/2019 to 12/31/19 | 100.2 | 101.39 | 100.09 | 100.68 | 104.02 | 101 | 102.3 | 100.53 |
| | 2021 | 4/1/2021 to 5/31/21 | 88 | 88.86 | 88.13 | 87.67 | 87.23 | 87.26 | 87.37 | 87.29 |
| | | | B1A | B1B | B2A | B2B | B3A | B3B | B4A | B4B |
| Amoxicillin | 2018 | 9/1/2018 | 100.16 | 97.44 | 100.96 | 100.04 | 101.57 | 99.31 | 98.74 | 102.42 |
| | 2019 | 12/1/2018 | 102.08 | 98.58 | 101.51 | 100.02 | 99.68 | 97.11 | 98.97 | 93.72 |
| | 2021 | 7/1/2021 | 96.42 | 93.83 | 94.64 | 94.25 | 89.97 | 95.26 | 95.23 | 95.03 |

## Supplemental Table 6. Dissolution results

| Approx. analysis date | Sample | Code | Product Name | Strength | units | Dose form | Treatment | Control type | Rdn Dose | % Dissolved | | USP Result |
|---|---|---|---|---|---|---|---|---|---|---|---|---|
| 8/1/2018 | 2018 | C1A | Ibuprofen | 400 | mg | Tablet | Non-irradiated | JSC | Control | 100.64% | 1.32 | Pass |
| | 2018 | C1B | Ibuprofen | 400 | mg | Tablet | Non-irradiated | JSC | Control | 100.97% | 0.95 | Pass |
| | 2018 | C2A | Ibuprofen | 400 | mg | Tablet | Non-irradiated | Traveling | Control | 100.38% | 1.52 | Pass |
| | 2018 | C2B | Ibuprofen | 400 | mg | Tablet | Non-irradiated | Traveling | Control | 100.58% | 2.39 | Pass |
| | 2018 | C3A | Ibuprofen | 400 | mg | Tablet | Irradiation | | 0.5 Gy | 100.49% | 1.92 | Pass |
| | 2018 | C3B | Ibuprofen | 400 | mg | Tablet | Irradiation | | 0.5 Gy | 100.59% | 3.26 | Pass |
| | 2018 | C4A | Ibuprofen | 400 | mg | Tablet | Irradiation | | 1.0 Gy | 100.53% | 1.36 | Pass |
| | 2018 | C4B | Ibuprofen | 400 | mg | Tablet | Irradiation | | 1.0 Gy | 100.00% | 2.66 | Pass |
| 8/1/2018 | 2018 | A1A | Acetaminophen | 500 | mg | Tablet | Non-irradiated | JSC | Control | 99.51% | 1.1 | Pass |
| | 2018 | A1B | Acetaminophen | 500 | mg | Tablet | Non-irradiated | JSC | Control | 100.71% | 3.56 | Pass |
| | 2018 | A2A | Acetaminophen | 500 | mg | Tablet | Non-irradiated | Traveling | Control | 100.12% | 2.96 | Pass |
| | 2018 | A2B | Acetaminophen | 500 | mg | Tablet | Non-irradiated | Traveling | Control | 100.77% | 4.48 | Pass |
| | 2018 | A3A | Acetaminophen | 500 | mg | Tablet | Irradiation | | 0.5 Gy | 102.75% | 4.01 | Pass |
| | 2018 | A3B | Acetaminophen | 500 | mg | Tablet | Irradiation | | 0.5 Gy | 100.85% | 2.19 | Pass |
| | 2018 | A4A | Acetaminophen | 500 | mg | Tablet | Irradiation | | 1 Gy | 99.51% | 2.81 | Pass |
| | 2018 | A4B | Acetaminophen | 500 | mg | Tablet | Irradiation | | 1 Gy | 95.45% | 4.47 | Pass |
| 8/1/2018 | 2018 | E1A | Promethazine | 25 | mg | Tablet | Non-irradiated | JSC | Control | 98.48% | 0.92 | Pass |
| | 2018 | E1B | Promethazine | 25 | mg | Tablet | Non-irradiated | JSC | Control | 98.38% | 0.58 | Pass |
| | 2018 | E2A | Promethazine | 25 | mg | Tablet | Non-irradiated | Traveling | Control | 98.21% | 2.13 | Pass |
| | 2018 | E2B | Promethazine | 25 | ma | Tablet | Non-irradiated | Traveling | Control | 98.69% | 1.35 | Pass |
| | 2018 | E3A | Promethazine | 25 | mg | Tablet | Irradiation | | 0.5Gy | 98.12% | 1.69 | Pass |
| | 2018 | E3B | Promethazine | 25 | mg | Tablet | Irradiation | | 0.5Gv | 98.58% | 0.8 | Pass |
| | 2018 | E4A | Promethazine | 25 | mg | Tablet | Irradiation | | 1.0Gy | 98.41% | 1.47 | Pass |
| | 2018 | E4B | Promethazine | 25 | mg | Tablet | Irradiation | | 1.0Gy | 98.48% | 0.62 | Pass |
| 8/1/2018 | 2018 | B1A | Amoxicillin | 500 | mg | Capsules | Non-irradiated | JSC | Control | 100.16% | 5.78 | Pass |
| | 2018 | B1B | Amoxicillin | 500 | mg | Capsules | Non-irradiated | JSC | Control | 97.44% | 5.06 | Pass |
| | 2018 | B2A | Amoxicillin | 500 | mg | Capsules | Non-irradiated | Traveling | Control | 100.96% | 4.63 | Pass |
| | 2018 | B2B | Amoxicillin | 500 | mg | Capsules | Non-irradiated | Traveling | Control | 100.04% | 4.7 | Pass |
| | 2018 | B3A | Amoxicillin | 500 | mg | Capsules | Irradiation | | 0.5Gy | 101.57% | 6.17 | Pass |
| | 2018 | B3B | Amoxicillin | 500 | mg | Capsules | Irradiation | | 0.5Gv | 99.31% | 5.46 | Pass |
| | 2018 | B4A | Amoxicillin | 500 | mg | Capsules | Irradiation | | 1.0Gv | 98.74% | 4.53 | Pass |
| | 2018 | 84B | Amoxicillin | 500 | mg | Capsules | Irradiation | | 1.0Gy | 102.42% | 2.49 | Pass |

| | | | | | | | | | | | | |
|---|---|---|---|---|---|---|---|---|---|---|---|---|
| 11/1/2019 to 12/31/19 | 2019 | C1A | Ibuprofen | 400 | mg | Tablet | Non-irradiated | JSC | Control | 98.23% | 0.20% | Pass |
| | 2019 | C1B | Ibuprofen | 400 | mg | Tablet | Non-irradiated | JSC | Control | 98.17% | 0.16% | Pass |
| | 2019 | C2A | Ibuprofen | 400 | mg | Tablet | Non-irradiated | Traveling | Control | 98.11% | 0.00% | Pass |
| | 2019 | C2B | Ibuprofen | 400 | mg | Tablet | Non-irradiated | Traveling | Control | 98.55% | 0.38% | Pass |
| | 2019 | C3A | Ibuprofen | 400 | mg | Tablet | Irradiation | | 0.5 Gy | 98.74% | 0.40% | Pass |
| | 2019 | C3B | Ibuprofen | 400 | mg | Tablet | Irradiation | | 0.5 Gy | 98.86% | 0.42% | Pass |
| | 2019 | C4A | Ibuprofen | 400 | mg | Tablet | Irradiation | | 1.0 Gy | 98.99% | 0.71% | Pass |
| | 2019 | C4B | Ibuprofen | 400 | mg | Tablet | Irradiation | | 1.0 Gy | 99.05% | 0.86% | Pass |
| 11/1/2019 to 12/31/19 | 2019 | A1A | Acetaminophen | 500 | mg | Tablet | Non-irradiated | JSC | Control | 102.54% | 1.07% | Pass |
| | 2019 | A1B | Acetaminophen | 500 | mg | Tablet | Non-irradiated | JSC | Control | 100.40% | 1.24% | Pass |
| | 2019 | A2A | Acetaminophen | 500 | mg | Tablet | Non-irradiated | Traveling | Control | 101.09% | 1.49% | Pass |
| | 2019 | A2B | Acetaminophen | 500 | mg | Tablet | Non-irradiated | Traveling | Control | 99.47% | 2.08% | Pass |
| | 2019 | A3A | Acetaminophen | 500 | mg | Tablet | Irradiation | | 0.5 Gy | 100.49% | 1.67% | Pass |
| | 2019 | A3B | Acetaminophen | 500 | mg | Tablet | Irradiation | | 0.5 Gy | 101.19% | 0.86% | Pass |
| | 2019 | A4A | Acetaminophen | 500 | mg | Tablet | Irradiation | | 1 Gy | 100.43% | 1.56% | Pass |
| | 2019 | A4B | Acetaminophen | 500 | mg | Tablet | Irradiation | | 1 Gy | 100.74% | 2.08% | Pass |
| 11/1/2019 to 12/31/19 | 2019 | E1A | Promethazine | 25 | mg | Tablet | Non-irradiated | JSC | Control | 103.46% | 0.53% | Pass |
| | 2019 | E1B | Promethazine | 25 | mg | Tablet | Non-irradiated | JSC | Control | 103.95% | 0.68% | Pass |
| | 2019 | E2A | Promethazine | 25 | mg | Tablet | Non-irradiated | Traveling | Control | 102.94% | 0.46% | Pass |
| | 2019 | E2B | Promethazine | 25 | ma | Tablet | Non-irradiated | Traveling | Control | 103.93% | 0.36% | Pass |
| | 2019 | E3A | Promethazine | 25 | mg | Tablet | Irradiation | | 0.5Gy | 103.90% | 0.32% | Pass |
| | 2019 | E3B | Promethazine | 25 | mg | Tablet | Irradiation | | 0.5Gv | 104.03% | 0.59% | Pass |
| | 2019 | E4A | Promethazine | 25 | mg | Tablet | Irradiation | | 1.0Gy | 103.50% | 0.51% | Pass |
| | 2019 | E4B | Promethazine | 25 | mg | Tablet | Irradiation | | 1.0Gy | 103.46% | 0.53% | Pass |
| 11/1/2019 to 12/31/19 | 2019 | B1A | Amoxicillin | 500 | mg | Capsules | Non-irradiated | JSC | Control | 93.43% | 0.0212 | Pass |
| | 2019 | B1B | Amoxicillin | 500 | mg | Capsules | Non-irradiated | JSC | Control | 92.18% | 0.0453 | Pass |
| | 2019 | B2A | Amoxicillin | 500 | mg | Capsules | Non-irradiated | Traveling | Control | 89.69% | 0.0316 | Pass |
| | 2019 | B2B | Amoxicillin | 500 | mg | Capsules | Non-irradiated | Traveling | Control | 92.80% | 0.0165 | Pass |
| | 2019 | B3A | Amoxicillin | 500 | mg | Capsules | Irradiation | | 0.5Gy | 91.25% | 0.0389 | Pass |
| | 2019 | B3B | Amoxicillin | 500 | mg | Capsules | Irradiation | | 0.5Gv | 91.05% | 0.0543 | Pass |
| | 2019 | B4A | Amoxicillin | 500 | mg | Capsules | Irradiation | | 1.0Gv | 86.13% | 0.0277 | Pass |
| | 2019 | 84B | Amoxicillin | 500 | mg | Capsules | Irradiation | | 1.0Gy | 88.59% | 0.0518 | Pass |

| | | | | | | | | | | | | |
|---|---|---|---|---|---|---|---|---|---|---|---|---|
| | 2021 | C1A | Ibuprofen | 400 | mg | Tablet | Non-irradiated | JSC | Control | Insufficent sample, NR | | |

| | | | | | | | | | | |
|---|---|---|---|---|---|---|---|---|---|---|
| 4/1/2021 to 5/31/21 | 2021 | C1B | Ibuprofen | 400 | mg | Tablet | Non-irradiated | JSC | Control | Insufficent sample, NR |
| | 2021 | C2A | Ibuprofen | 400 | mg | Tablet | Non-irradiated | Traveling | Control | Insufficent sample, NR |
| | 2021 | C2B | Ibuprofen | 400 | mg | Tablet | Non-irradiated | Traveling | Control | Insufficent sample, NR |
| | 2021 | C3A | Ibuprofen | 400 | mg | Tablet | Irradiation | | 0.5 Gy | Insufficent sample, NR |
| | 2021 | C3B | Ibuprofen | 400 | mg | Tablet | Irradiation | | 0.5 Gy | Insufficent sample, NR |
| | 2021 | C4A | Ibuprofen | 400 | mg | Tablet | Irradiation | | 1.0 Gy | Insufficent sample, NR |
| | 2021 | C4B | Ibuprofen | 400 | mg | Tablet | Irradiation | | 1.0 Gy | Insufficent sample, NR |
| 4/1/2021 to 5/31/21 | 2021 | A1A | Acetaminophen | 500 | mg | Tablet | Non-irradiated | JSC | Control | Insufficent sample, NR |
| | 2021 | A1B | Acetaminophen | 500 | mg | Tablet | Non-irradiated | JSC | Control | Insufficent sample, NR |
| | 2021 | A2A | Acetaminophen | 500 | mg | Tablet | Non-irradiated | Traveling | Control | Insufficent sample, NR |
| | 2021 | A2B | Acetaminophen | 500 | mg | Tablet | Non-irradiated | Traveling | Control | Insufficent sample, NR |
| | 2021 | A3A | Acetaminophen | 500 | mg | Tablet | Irradiation | | 0.5 Gy | Insufficent sample, NR |
| | 2021 | A3B | Acetaminophen | 500 | mg | Tablet | Irradiation | | 0.5 Gy | Insufficent sample, NR |
| | 2021 | A4A | Acetaminophen | 500 | mg | Tablet | Irradiation | | 1 Gy | Insufficent sample, NR |
| | 2021 | A4B | Acetaminophen | 500 | mg | Tablet | Irradiation | | 1 Gy | Insufficent sample, NR |
| 4/1/2021 to 5/31/21 | 2021 | E1A | Promethazine | 25 | mg | Tablet | Non-irradiated | JSC | Control | Insufficent sample, NR |
| | 2021 | E1B | Promethazine | 25 | mg | Tablet | Non-irradiated | JSC | Control | Insufficent sample, NR |
| | 2021 | E2A | Promethazine | 25 | mg | Tablet | Non-irradiated | Traveling | Control | Insufficent sample, NR |
| | 2021 | E2B | Promethazine | 25 | ma | Tablet | Non-irradiated | Traveling | Control | Insufficent sample, NR |
| | 2021 | E3A | Promethazine | 25 | mg | Tablet | Irradiation | | 0.5Gy | Insufficent sample, NR |
| | 2021 | E3B | Promethazine | 25 | mg | Tablet | Irradiation | | 0.5Gv | Insufficent sample, NR |
| | 2021 | E4A | Promethazine | 25 | mg | Tablet | Irradiation | | 1.0Gy | Insufficent sample, NR |
| | 2021 | E4B | Promethazine | 25 | mg | Tablet | Irradiation | | 1.0Gy | Insufficent sample, NR |
| 4/1/2021 to 5/31/21 | 2021 | B1A | Amoxicillin | 500 | mg | Capsules | Non-irradiated | JSC | Control | Insufficent sample, NR |
| | 2021 | B1B | Amoxicillin | 500 | mg | Capsules | Non-irradiated | JSC | Control | Insufficent sample, NR |
| | 2021 | B2A | Amoxicillin | 500 | mg | Capsules | Non-irradiated | Traveling | Control | Insufficent sample, NR |
| | 2021 | B2B | Amoxicillin | 500 | mg | Capsules | Non-irradiated | Traveling | Control | Insufficent sample, NR |
| | 2021 | B3A | Amoxicillin | 500 | mg | Capsules | Irradiation | | 0.5Gy | Insufficent sample, NR |
| | 2021 | B3B | Amoxicillin | 500 | mg | Capsules | Irradiation | | 0.5Gv | Insufficent sample, NR |
| | 2021 | B4A | Amoxicillin | 500 | mg | Capsules | Irradiation | | 1.0Gv | Insufficent sample, NR |
| | 2021 | 84B | Amoxicillin | 500 | mg | Capsules | Irradiation | | 1.0Gy | Insufficent sample, NR |

NR: no results

## Supplemental Table 7. Impurities Acetaminophen

| Sample | Peak | Report-peak | Retention_time (Min) | A1A | A1B | A2A | A2B | A3A | A3B | A4A | A4B |
|---|---|---|---|---|---|---|---|---|---|---|---|
| 2018 | 1 | P-Aminophenol | ND | ND | ND | ND | ND | ND | ND | ND | ND |
| 2018 | 2 | Acetaminophen | 1.8 | 99.53 | 99.53 | 99.49 | 99.55 | 99.54 | 99.52 | 99.55 | 99.51 |
| 2018 | 3 | ND | ND | ND | ND | ND | ND | ND | ND | ND | ND |
| 2018 | 4 | Unk3 | 3.022 | 0.01 | 0.01 | 0.01 | 0.01 | 0.01 | 0.01 | 0.01 | 0.01 |
| 2018 | 5 | Unk4 | 3.109 | 0.01 | 0.01 | 0.01 | 0.01 | 0.01 | 0.01 | 0.01 | 0.01 |
| 2018 | 6 | Unk5 | 3.738 | 0.11 | 0.12 | 0.15 | 0.11 | 0.11 | 0.12 | 0.1 | 0.13 |
| 2018 | 7 | Unk6 | 4.946 | 0.03 | 0.03 | 0.04 | 0.03 | 0.03 | 0.03 | 0.03 | 0.04 |
| 2018 | 8 | Unk7 | 6.241 | ND | 0.01 | ND | ND | ND | 0.01 | ND | 0.01 |
| 2018 | 9 | Unk8 | 7.369 | 0.02 | 0.02 | 0.02 | 0.02 | 0.02 | 0.03 | 0.02 | 0.03 |
| 2018 | 10 | ND | 8.052 | ND | ND | ND | ND | ND | ND | ND | ND |
| 2018 | 11 | Unk9 | 8.32 | 0.15 | 0.15 | 0.15 | 0.15 | 0.14 | 0.15 | 0.15 | 0.13 |
| 2018 | 12 | Unk10 | 8.935 | 0.02 | 0.02 | 0.02 | 0.02 | 0.02 | 0.02 | 0.02 | 0.02 |
| 2018 | 13 | Unk11 | 9.4 | 0.07 | 0.07 | 0.07 | 0.07 | 0.07 | 0.07 | 0.07 | 0.07 |
| 2018 | 14 | Unk12 | 9.872 | 0.02 | 0.02 | 0.02 | 0.02 | 0.02 | 0.02 | 0.02 | 0.02 |
| 2018 | 15 | Unk13 | 10.875 | 0.01 | 0.01 | 0.01 | ND | 0.01 | ND | ND | 0.01 |
| 2018 | 16 | Unk14 | 10.937 | 0.01 | ND | 0.01 | 0.01 | 0.01 | 0.01 | 0.01 | 0.01 |
| 2018 | 17 | ND | ND | ND | ND | ND | ND | ND | ND | ND | ND |
| | SUM | NMT | 0.6 | 0.46 | 0.47 | 0.51 | 0.45 | 0.45 | 0.48 | 0.44 | 0.49 |
| 2019 | 1 | P-Aminophenol | ND | ND | ND | ND | ND | ND | ND | ND | ND |
| 2019 | 2 | APAP not reported | 1.751 | 99.777 | 99.77 | 99.764 | 99.772 | 99.771 | 99.764 | 99.78 | 99.767 |
| 2019 | 3 | ND | ND | ND | ND | ND | ND | ND | ND | ND | ND |
| 2019 | 4 | ND | ND | ND | ND | ND | ND | ND | ND | ND | ND |
| 2019 | 5 | ND | ND | ND | ND | ND | ND | ND | ND | ND | ND |
| 2019 | 6 | Unk2 | 3.665 | 0.02 | 0.02 | 0.02 | 0.02 | 0.02 | 0.02 | 0.02 | 0.02 |
| 2019 | 7 | Unk3 | 4.817 | 0.02 | 0.03 | 0.03 | 0.025 | 0.02 | 0.02 | 0.02 | 0.02 |
| 2019 | 8 | Unk4 | 6.241 | ND | ND | ND | ND | ND | 0.01 | ND | 0.01 |
| 2019 | 9 | ND | ND | ND | ND | ND | ND | ND | ND | ND | ND |
| 2019 | 10 | Unk5 | 8.052 | 0.163 | 0.16 | 0.166 | 0.163 | 0.166 | 0.163 | 0.16 | 0.163 |
| 2019 | 11 | ND | ND | ND | ND | ND | ND | ND | ND | ND | ND |
| 2019 | 12 | ND | ND | ND | ND | ND | ND | ND | ND | ND | ND |
| 2019 | 13 | Unk6 | 9.34 | 0.02 | 0.02 | 0.02 | 0.02 | 0.023 | 0.023 | 0.02 | 0.02 |
| 2019 | 14 | ND | ND | ND | ND | ND | ND | ND | ND | ND | ND |
| 2019 | 15 | ND | ND | ND | ND | ND | ND | ND | ND | ND | ND |
| 2019 | 16 | ND | ND | ND | ND | ND | ND | ND | ND | ND | ND |
| 2019 | 17 | ND | ND | ND | ND | ND | ND | ND | ND | ND | ND |
| | SUM | NMT | 0.6 | 0.223 | 0.23 | 0.236 | 0.228 | 0.229 | 0.236 | 0.22 | 0.233 |
| 2021 | 1 | P-Aminophenol | ND | ND | ND | ND | ND | ND | ND | ND | ND |
| 2021 | 2 | APAP Not reported | NA | 99.584 | 99.583 | 99.583 | 99.573 | 99.557 | 99.633 | 99.56 | 99.576 |
| 2021 | 3 | Unk2 | 2.69 | 0.033 | 0.04 | 0.03 | 0.03 | 0.03 | ND | 0.05 | 0.03 |
| 2021 | 4 | Unk3 | 2.93 | 0.027 | 0.023 | 0.023 | 0.023 | 0.023 | ND | 0.023 | 0.027 |
| 2021 | 5 | ND | ND | ND | ND | ND | ND | ND | ND | ND | ND |
| 2021 | 6 | Unk4 | 3.6 | 0.1 | 0.1 | 0.1 | 0.1 | 0.1 | 0.09 | 0.1 | 0.1 |
| 2021 | 7 | Unk5 | 4.69 | 0.05 | 0.05 | 0.05 | 0.05 | 0.05 | 0.05 | 0.053 | 0.05 |
| 2021 | 8 | ND | ND | ND | ND | ND | ND | ND | ND | ND | ND |
| 2021 | 9 | Unk6 | 7.83 | 0.123 | 0.12 | 0.12 | 0.12 | 0.13 | 0.13 | 0.12 | 0.123 |
| 2021 | 10 | ND | ND | ND | ND | ND | ND | ND | ND | ND | ND |
| 2021 | 11 | ND | ND | ND | ND | ND | ND | ND | ND | ND | ND |
| 2021 | 12 | ND | ND | ND | ND | ND | ND | ND | ND | ND | ND |
| 2021 | 13 | Unk7 | 8.97 | 0.013 | 0.027 | 0.027 | 0.027 | 0.03 | 0.037 | 0.027 | 0.027 |
| 2021 | 14 | Unk8 | 9.38 | 0.03 | 0.017 | 0.027 | 0.027 | 0.027 | 0.03 | 0.027 | 0.027 |
| 2021 | 15 | Unk9 | 10.38 | 0.01 | 0.01 | 0.01 | 0.02 | 0.02 | ND | 0.01 | 0.01 |
| 2021 | 16 | ND | ND | ND | ND | ND | ND | ND | ND | ND | ND |
| 2021 | 17 | 1Unk0 | 11.5 | 0.03 | 0.03 | 0.03 | 0.03 | 0.033 | 0.03 | 0.03 | 0.03 |
| | SUM | NMT | 0.6 | 0.386 | 0.387 | 0.387 | 0.397 | 0.41 | 0.337 | 0.41 | 0.394 |

## Supplemental Table 8. Impurities ibuprofen

| Sample | Peak | Report-peak | Retention_time (Min) | C1A | C1B | C2A | C2B | C3A | C3B | C4A | C4B |
|---|---|---|---|---|---|---|---|---|---|---|---|
| 2018 | 1 | Unknown1 | 0.25 | ND | ND | ND | 0.86 | ND | ND | ND | ND |
| 2018 | 2 | Related_J | 0.41 | 1.55 | 1.54 | 1.57 | 1.51 | 1.57 | 1.57 | 1.55 | 1.57 |
| 2018 | 3 | ND | 0.51 | ND | ND | ND | ND | ND | ND | ND | ND |
| 2018 | 4 | ND | 0.548 | ND | ND | ND | ND | ND | ND | ND | ND |
| 2018 | 5 | Unknown3 | 0.622 | 0.17 | 0.13 | 0.16 | 0.12 | 0.15 | 0.23 | 0.14 | 0.17 |
| 2018 | 6 | Ibuprofen | 0.742 | 98.06 | 98.06 | 98.01 | 97.24 | 98.02 | 97.93 | 98.05 | 98 |
| 2018 | 7 | Related C | 1.011 | 0.22 | 0.27 | 0.25 | 0.27 | 0.26 | 0.27 | 0.26 | 0.25 |

| | | | | | | | | | | | |
|---|---|---|---|---|---|---|---|---|---|---|---|
| 2019 | 1 | ND | ND | ND | ND | ND | ND | ND | ND | ND | ND |
| 2019 | 2 | Related J | 0.351 | 1.79 | 1.77 | 1.82 | 1.78 | 1.71 | 1.77 | 1.81 | 1.76 |
| 2019 | 3 | ND | 0.51 | ND | ND | ND | ND | ND | ND | ND | ND |
| 2019 | 4 | Unknown | 0.548 | 0.12 | 0.115 | 0.125 | 0.12 | 0.11 | 0.11 | 0.12 | 0.12 |
| 2019 | 5 | Unknown3 | 0.622 | ND | ND | ND | ND | ND | ND | ND | ND |
| 2019 | 6 | Ibu not reported | 0.73 | 97.8 | 97.775 | 97.705 | 97.75 | 97.85 | 97.79 | 97.73 | 97.79 |
| 2019 | 7 | Related C | 0.902 | 0.29 | 0.34 | 0.35 | 0.35 | 0.33 | 0.33 | 0.34 | 0.33 |

| | | | | | | | | | | | |
|---|---|---|---|---|---|---|---|---|---|---|---|
| 2021 | 1 | | ND | ND | ND | ND | ND | ND | ND | ND | ND |
| 2021 | 2 | Related J | 0.351 | 0.12 | 0.12 | 0.12 | 0.12 | 0.13 | 0.13 | 0.11 | 0.12 |
| 2021 | 3 | Unknown2 | 0.51 | 0.47 | 0.45 | 0.42 | 0.47 | 0.51 | 0.46 | 0.39 | 0.49 |
| 2021 | 4 | Unknown1 | 0.548 | 0.12 | 0.115 | 0.125 | 0.12 | 0.11 | 0.11 | 0.12 | 0.12 |
| 2021 | 5 | Unknown3 | 0.62 | 1.91 | 1.9 | 1.87 | 1.9 | 1.98 | 1.89 | 1.82 | 1.97 |
| 2021 | 6 | Ibu not reported | 0.73 | 97.07 | 97.065 | 97.085 | 97.01 | 96.88 | 97.02 | 97.2 | 96.97 |
| 2021 | 7 | Related C | 0.902 | 0.31 | 0.35 | 0.38 | 0.38 | 0.39 | 0.39 | 0.36 | 0.33 |

## Supplemental Table 9. Impurities Amoxicillin

| Sample | Peak | Report-peak | Retention_time (Min) | B1A | B1B | B2A | B2B | B3A | B3B | B4A | B4B |
|---|---|---|---|---|---|---|---|---|---|---|---|
| 2018 | 1 | Related_I | 0.52 | 0.53 | 0.48 | 0.49 | 0.49 | 0.46 | 0.47 | 0.42 | 0.45 |
| 2018 | 2 | 2 | 0.602 | 0.05 | 0.05 | 0.05 | 0.05 | 0.05 | 0.05 | 0.05 | 0.05 |
| 2018 | 3 | 3 | 0.686 | 0.11 | 0.11 | 0.11 | 0.12 | 0.13 | 0.13 | 0.16 | 0.13 |
| 2018 | 4 | 4 | 1.158 | 0.05 | 0.05 | 0.05 | 0.05 | 0.05 | 0.05 | ND | 0.04 |
| 2018 | 5 | Related_D | 1.322 | 0.22 | 0.23 | 0.22 | 0.23 | 0.26 | 0.24 | 0.34 | 0.26 |
| 2018 | 6 | Related_A | 1.945 | 0.44 | 0.45 | 0.44 | 0.45 | 0.49 | 0.47 | 0.61 | 0.48 |
| 2018 | 7 | Related_B | 2.26 | ND | ND | ND | ND | ND | ND | ND | ND |
| 2018 | 8 | Amoxicillin | 2.885 | 95.55 | 95.51 | 95.73 | 95.66 | 95.39 | 95.49 | 94.74 | 95.61 |
| 2018 | 9 | 9 | 4.547 | 0.04 | 0.04 | 0.04 | 0.04 | 0.04 | 0.04 | 0.05 | 0.07 |
| 2018 | 10 | 10 | 5.497 | 0.04 | 0.04 | 0.04 | 0.04 | 0.04 | 0.04 | ND | ND |
| 2018 | 11 | 11 | 5.737 | 0.36 | 0.36 | 0.36 | 0.36 | 0.34 | 0.36 | 0.31 | 0.33 |
| 2018 | 12 | 12 | 6.051 | 0.05 | 0.05 | 0.04 | 0.04 | 0.05 | 0.05 | 0.05 | 0.04 |
| 2018 | 13 | 13 | 6.151 | 0.41 | 0.42 | 0.4 | 0.4 | 0.44 | 0.42 | 0.54 | 0.43 |
| 2018 | 14 | 14 | 6.758 | 0.05 | 0.05 | 0.05 | 0.05 | 0.05 | 0.05 | ND | 0.04 |
| 2018 | 15 | 15 | 7.453 | 0.03 | 0.03 | 0.03 | 0.03 | 0.04 | 0.04 | 0.05 | ND |
| 2018 | 16 | Related_E | 7.592 | 0.48 | 0.49 | 0.48 | 0.48 | 0.51 | 0.49 | 0.6 | 0.49 |
| 2018 | 17 | Related_G | 8.139 | 0.11 | 0.12 | 0.12 | 0.11 | 0.13 | 0.13 | 0.17 | 0.13 |
| 2018 | 18 | 18 | 9.062 | 0.06 | 0.06 | 0.06 | 0.06 | 0.06 | 0.06 | 0.07 | 0.06 |
| 2018 | 19 | Related_C | 9.167 | 0.04 | 0.04 | 0.04 | 0.04 | 0.05 | 0.05 | 0.08 | 0.05 |
| 2018 | 20 | 20 | 9.492 | 0.06 | 0.07 | 0.06 | 0.06 | 0.08 | 0.08 | 0.11 | 0.08 |
| 2018 | 21 | 21 | 10.284 | 0.06 | ND | ND | ND | ND | ND | ND | ND |
| 2018 | 22 | 22 | 10.536 | 0.04 | 0.04 | 0.04 | 0.04 | 0.04 | 0.04 | ND | 0.04 |
| 2018 | 23 | Related_H | 10.708 | 0.69 | 0.71 | 0.7 | 0.69 | 0.76 | 0.72 | 0.98 | 0.72 |
| 2018 | 24 | 24 | 10.815 | 0.13 | 0.14 | 0.13 | 0.12 | 0.14 | 0.13 | 0.19 | 0.14 |
| 2018 | 25 | 25 | 11.112 | 0.11 | 0.12 | 0.11 | 0.1 | 0.12 | 0.12 | 0.17 | 0.12 |
| 2018 | 26 | 26 | 11.188 | 0.03 | 0.04 | 0.03 | ND | ND | ND | ND | ND |
| 2018 | 27 | 27 | 11.437 | 0.06 | 0.06 | 0.05 | 0.04 | 0.05 | 0.05 | 0.07 | 0.05 |
| 2018 | 28 | Dimer | 11.899 | 0.07 | 0.08 | 0.06 | 0.08 | 0.09 | 0.08 | 0.12 | 0.09 |
| 2018 | 29 | 29 | 12.16 | 0.03 | 0.04 | ND | 0.04 | 0.04 | 0.04 | 0.06 | ND |
| 2018 | 30 | 30 | 12.397 | 0.07 | 0.07 | ND | 0.05 | 0.04 | 0.04 | ND | 0.04 |
| 2018 | 31 | 31 | 13.267 | 0.04 | 0.04 | 0.03 | 0.04 | 0.05 | 0.04 | 0.06 | 0.05 |

| | | | | | | | | | | | |
|---|---|---|---|---|---|---|---|---|---|---|---|
| 2019 | 1 | Related I | 0.504 | 0.46 | 0.47 | 0.46 | 0.46 | 0.46 | 0.47 | 0.51 | 0.51 |
| 2019 | 2 | NR | NA | NA | NA | NA | NA | NA | NA | NA | NA |
| 2019 | 3 | NR | NA | NA | NA | NA | NA | NA | NA | NA | NA |
| 2019 | 4 | NR | NA | NA | NA | NA | NA | NA | NA | NA | NA |
| 2019 | 5 | Related D | 1.322 | 0.32 | 0.33 | 0.29 | 0.29 | 0.28 | 0.28 | ND | ND |
| 2019 | 6 | Related A | 1.945 | 0.42 | 0.43 | 0.42 | 0.42 | 0.41 | 0.43 | 0.38 | 0.43 |
| 2019 | 7 | Related B | 2.26 | ND | ND | ND | ND | ND | ND | ND | ND |
| 2019 | 8 | Amoxicillin | 2.78 | 98.08 | 98.01 | 98.08 | 98.09 | 98.09 | 98.08 | 98.21 | 98.17 |
| 2019 | 9 | NR | NA | NA | NA | NA | NA | NA | NA | NA | NA |
| 2019 | 10 | NR | NA | NA | NA | NA | NA | NA | NA | NA | NA |
| 2019 | 11 | NR | NA | NA | NA | NA | NA | NA | NA | NA | NA |
| 2019 | 12 | NR | NA | NA | NA | NA | NA | NA | NA | NA | NA |
| 2019 | 13 | NR | NA | NA | NA | NA | NA | NA | NA | NA | NA |
| 2019 | 14 | NR | NA | NA | NA | NA | NA | NA | NA | NA | NA |
| 2019 | 15 | NR | NA | NA | NA | NA | NA | NA | NA | NA | NA |
| 2019 | 16 | Related E | 7.782 | 0.59 | 0.61 | 0.6 | 0.59 | 0.59 | 0.59 | 0.72 | 0.72 |
| 2019 | 17 | Related G | 8.139 | ND | ND | ND | ND | ND | ND | ND | ND |
| 2019 | 18 | NR | NA | NA | NA | NA | NA | NA | NA | NA | NA |
| 2019 | 19 | Related C | 9.3 | ND | ND | ND | ND | ND | ND | ND | ND |
| 2019 | 20 | NR | NA | NA | NA | NA | NA | NA | NA | NA | NA |
| 2019 | 21 | NR | NA | NA | NA | NA | NA | NA | NA | NA | NA |

| | | | | | | | | | | | |
|---|---|---|---|---|---|---|---|---|---|---|---|
| 2019 | 22 | NR | NA | NA | NA | NA | NA | NA | NA | NA | NA |
| 2019 | 23 | Related H | 10.567 | 0.59 | 0.62 | 0.61 | 0.61 | 0.63 | 0.62 | 0.69 | 0.68 |
| 2019 | 24 | NR | NA | NA | NA | NA | NA | NA | NA | NA | NA |
| 2019 | 25 | NR | NA | NA | NA | NA | NA | NA | NA | NA | NA |
| 2019 | 26 | NR | NA | NA | NA | NA | NA | NA | NA | NA | NA |
| 2019 | 27 | NR | NA | NA | NA | NA | NA | NA | NA | NA | NA |
| 2019 | 28 | Dimer | 11.899 | ND | ND | ND | ND | ND | ND | ND | ND |
| 2019 | 29 | NR | NA | NA | NA | NA | NA | NA | NA | NA | NA |
| 2019 | 30 | NR | NA | NA | NA | NA | NA | NA | NA | NA | NA |
| 2019 | 31 | NR | NA | NA | NA | NA | NA | NA | NA | NA | NA |

| | | | | | | | | | | | |
|---|---|---|---|---|---|---|---|---|---|---|---|
| 2021 | 1 | Related I | 0.504 | 0.48 | 0.53 | 0.5 | 0.51 | 0.54 | 0.5 | 0.52 | 0.53 |
| 2021 | 2 | NR | NA | NA | NA | NA | NA | NA | NA | NA | NA |
| 2021 | 3 | NR | NA | NA | NA | NA | NA | NA | NA | NA | NA |
| 2021 | 4 | NR | NA | NA | NA | NA | NA | NA | NA | NA | NA |
| 2021 | 5 | Related D | 1.322 | 0.38 | 0.33 | 0.37 | 0.37 | 0.32 | 0.38 | 0.36 | 0.36 |
| 2021 | 6 | Related A | 1.945 | 0.48 | 0.46 | 0.47 | 0.44 | 0.47 | 0.48 | 0.5 | 0.5 |
| 2021 | 7 | Related B | 2.26 | ND | ND | ND | ND | ND | ND | ND | ND |
| 2021 | 8 | Amoxicillin | 2.81 | 96.6 | 96.75 | 96.62 | 96.74 | 96.86 | 96.62 | 96.67 | 96.69 |
| 2021 | 9 | NR | NA | NA | NA | NA | NA | NA | NA | NA | NA |
| 2021 | 10 | NR | NA | NA | NA | NA | NA | NA | NA | NA | NA |
| 2021 | 11 | NR | NA | NA | NA | NA | NA | NA | NA | NA | NA |
| 2021 | 12 | NR | NA | NA | NA | NA | NA | NA | NA | NA | NA |
| 2021 | 13 | NR | NA | NA | NA | NA | NA | NA | NA | NA | NA |
| 2021 | 14 | NR | NA | NA | NA | NA | NA | NA | NA | NA | NA |
| 2021 | 15 | NR | NA | NA | NA | NA | NA | NA | NA | NA | NA |
| 2021 | 16 | Related E | 7.782 | 1.07 | 1.04 | 1.08 | 1.05 | 0.98 | 1.07 | 1.03 | 1.02 |
| 2021 | 17 | Related G | 8.139 | 0.19 | 0.18 | 0.19 | 0.19 | 0.17 | 0.2 | 0.2 | 0.2 |
| 2021 | 18 | NR | NA | NA | NA | NA | NA | NA | NA | NA | NA |
| 2021 | 19 | Related C | 9.3 | ND | ND | ND | ND | ND | ND | ND | ND |
| 2021 | 20 | NR | NA | NA | NA | NA | NA | NA | NA | NA | NA |
| 2021 | 21 | NR | NA | NA | NA | NA | NA | NA | NA | NA | NA |
| 2021 | 22 | NR | NA | NA | NA | NA | NA | NA | NA | NA | NA |
| 2021 | 23 | Related H | 10.567 | 0.65 | 0.59 | 0.63 | 0.6 | 0.55 | 0.64 | 0.59 | 0.58 |
| 2021 | 24 | NR | NA | NA | NA | NA | NA | NA | NA | NA | NA |
| 2021 | 25 | NR | NA | NA | NA | NA | NA | NA | NA | NA | NA |
| 2021 | 26 | NR | NA | NA | NA | NA | NA | NA | NA | NA | NA |
| 2021 | 27 | NR | NA | NA | NA | NA | NA | NA | NA | NA | NA |
| 2021 | 28 | Dimer | 11.899 | 0.15 | 0.12 | 0.14 | 0.1 | 0.11 | 0.11 | 0.13 | 0.12 |
| 2021 | 29 | NR | NA | NA | NA | NA | NA | NA | NA | NA | NA |
| 2021 | 30 | NR | NA | NA | NA | NA | NA | NA | NA | NA | NA |
| 2021 | 31 | NR | NA | NA | NA | NA | NA | NA | NA | NA | NA |

NR: Not reported.

## Supplemental Table 10. Impurities Promethazine

| Sample | Peak | Report-peak | Retention_time (Min) | E1A | E1B | E2A | E2B | E3A | E3B | E4A | E4B |
|---|---|---|---|---|---|---|---|---|---|---|---|
| 2018 | 1 | 1 | 1.71 | 0.08 | 0.03 | ND | ND | ND | ND | ND | ND |
| 2018 | 2 | 2 | 3.577 | 0.21 | 0.19 | 0.2 | 0.18 | 0.15 | 0.15 | 0.19 | 0.22 |
| 2018 | 3 | 3 | 3.651 | 0.05 | 0.05 | 0.06 | 0.05 | 0.03 | 0.06 | 0.07 | 0.05 |
| 2018 | 4 | 4 | 3.956 | 0.12 | 0.03 | 0.14 | 0.03 | 0.12 | 0.16 | 0.11 | 0.19 |
| 2018 | 5 | 5 | 4.026 | 0.02 | 0.12 | 0.03 | 0.13 | ND | 0.03 | 0.06 | 0.04 |
| 2018 | 6 | 6 | 4.734 | 0.04 | 0.04 | 0.04 | 0.04 | 0.06 | 0.05 | 0.05 | 0.06 |
| 2018 | 7 | 7 | 5.115 | 0.11 | 0.09 | 0.09 | 0.08 | 0.07 | 0.08 | 0.2 | 0.09 |
| 2018 | 8 | 8 | 5.274 | 0.1 | 0.1 | 0.1 | 0.1 | 0.1 | 0.09 | 0.11 | 0.03 |
| 2018 | 9 | Promethazine | 5.868 | 98.85 | 98.92 | 98.94 | 98.98 | 99.05 | 98.96 | 98.61 | 98.81 |
| 2018 | 10 | Related_B | 6.77 | 0.21 | 0.21 | 0.2 | 0.21 | 0.2 | 0.21 | 0.21 | 0.22 |
| 2018 | 11 | 11 | 8.791 | 0.18 | 0.2 | 0.2 | 0.19 | 0.18 | 0.17 | 0.21 | 0.23 |
| 2018 | 12 | 12 | 10.003 | 0.02 | 0.02 | ND | 0.03 | 0.03 | ND | ND | ND |
| 2018 | 13 | ND | ND | ND | ND | ND | ND | ND | ND | ND | ND |
| 2019 | 1 | ND | ND | ND | ND | ND | ND | ND | ND | ND | ND |
| 2019 | 2 | ND | ND | ND | ND | ND | ND | ND | ND | ND | ND |
| 2019 | 3 | 3 | 3.318 | 0.02 | 0.02 | 0.02 | 0.2 | 0.02 | 0.02 | 0.04 | 0.02 |
| 2019 | 4 | 4 | 3.516 | 0.03 | 0.03 | 0.03 | 0.3 | 0.03 | 0.03 | 0.1 | 0.03 |
| 2019 | 5 | 5 | 4.029 | 0.02 | 0.02 | 0.02 | 0.01 | 0.02 | 0.02 | 0.03 | 0.02 |
| 2019 | 6 | 6 | 4.571 | 0.03 | 0.03 | 0.02 | 0.025 | 0.02 | 0.02 | 0.16 | 0.02 |
| 2019 | 7 | 7 | 4.818 | 0.12 | 0.12 | 0.11 | 0.11 | 0.11 | 0.11 | 0.236 | 0.12 |
| 2019 | 8 | 8 | 4.945 | 0.33 | 0.32 | 0.33 | 0.33 | 0.33 | 0.33 | 0.566 | 0.32 |
| 2019 | 9 | Promethazine | 5.868 | 99 | 99.01 | 99.02 | 98.575 | 99.02 | 99.02 | 98.388 | 99.02 |
| 2019 | 10 | Related_B | 6.142 | 0.45 | 0.45 | 0.45 | 0.45 | 0.45 | 0.45 | 0.48 | 0.45 |
| 2019 | 11 | ND | ND | ND | ND | ND | ND | ND | ND | ND | ND |
| 2019 | 12 | ND | ND | ND | ND | ND | ND | ND | ND | ND | ND |
| 2019 | 13 | ND | ND | ND | ND | ND | ND | ND | ND | ND | ND |
| 2021 | 1 | ND | ND | ND | ND | ND | ND | ND | ND | ND | ND |
| 2021 | 2 | 2 | 1.28 | 0.03 | 0.03 | 0.03 | 0.03 | 0.03 | 0.027 | 0.03 | 0.03 |
| 2021 | 3 | 3 | 1.39 | 0.11 | 0.11 | 0.11 | 0.11 | 0.11 | 0.11 | 0.11 | 0.11 |
| 2021 | 4 | D | ND | ND | ND | ND | ND | ND | ND | ND | ND |
| 2021 | 5 | ND | ND | ND | ND | ND | ND | ND | ND | ND | ND |
| 2021 | 6 | ND | ND | ND | ND | ND | ND | ND | ND | ND | ND |
| 2021 | 7 | ND | ND | ND | ND | ND | ND | ND | ND | ND | ND |
| 2021 | 8 | ND | ND | ND | ND | ND | ND | ND | ND | ND | ND |
| 2021 | 9 | Promethazine | 2.2 | 99.79 | 99.78 | 99.79 | 99.78 | 99.78 | 99.793 | 99.77 | 99.783 |
| 2021 | 10 | Related_B | 2.7 | 0.03 | 0.03 | 0.03 | 0.03 | 0.03 | 0.027 | 0.03 | 0.03 |
| 2021 | 11 | ND | ND | ND | ND | ND | ND | ND | ND | ND | ND |
| 2021 | 12 | 12 | 4.1 | 0.03 | 0.03 | 0.03 | 0.03 | 0.03 | 0.033 | 0.04 | 0.037 |
| 2021 | 13 | 13 | 5.59 | 0.01 | 0.02 | 0.01 | 0.02 | 0.02 | 0.01 | 0.02 | 0.01 |

Empower™ 3 SOFTWARE

# Overlay Report

## Stacked Chromatograms

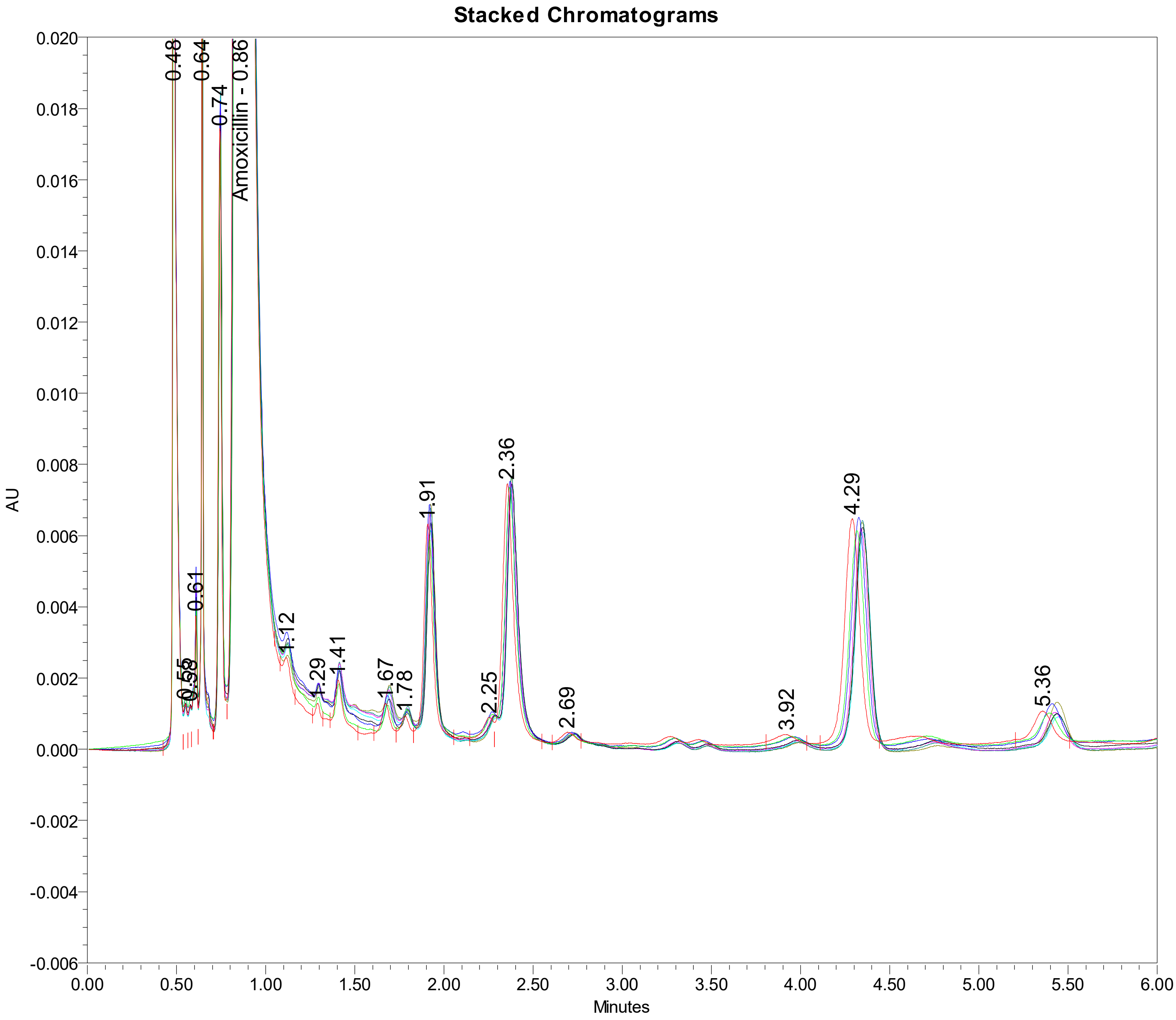


SampleName B4a; Vial 1:A,2; Injection 3
SampleName B4b; Vial 1:A,3; Injection 3
SampleName B1a; Vial 1:A,4; Injection 3
SampleName B1b; Vial 1:A,5; Injection 3
SampleName B3a; Vial 1:A,6; Injection 3
SampleName B3b; Vial 1:A,7; Injection 3
SampleName B2a; Vial 1:A,8; Injection 3
SampleName B2b; Vial 1:B,1; Injection 3

## 2. Scope

2.1 This method describes a procedure for determination of impurities in Ibuprofen Tablets, 400 mg. This method is adapted from and conforms to all procedures, tests and criteria described in the Organic Imputities section of the monograph for Ibuprofen Tablets in the USP41/NF16.

## 4. References

4.1 SOP-001 Quality Assurance Responsibilities
4.2 SOP-002 Document Control System
4.3 SOP-003 Good Documentation Practices
4.4 SOP-004 Records Management\
4.5 SOP-005 Nonconformances and Investigations
4.6 SOP-006 GMP Training Program
4.7 SOP-013 Material Specifications

4.8 SOP-016 Equipment Qualification
4.9 SOP-020 Equipment Calibration
4. SOP-024 Weighing on a Balance
4. Current USP/NF

## 7. Procedure

7.1 EQUIPMENT AND REAGENTS
- 7.1.1 Ibuprofen Reference Standard
- 7.1.2 Ibuprofen Related Compound C Reference Standard
- 7.1.3 Ibuprofen Related Compound J Reference Standard
- 7.1.4 Chloroacetic Acid, ACS Grade or equivalent
- 7.1.5 Ammonium Hydroxide, ACS Grade or equivalent
- 7.1.6 Water, HPLC Grade or equivalent
- 7.1.7 Acetonitrile, HPLC Grade or equivalent
- 7.1.8 Methanol, HPLC Grade or equivalent
- 7.1.9 Class A Glassware
- 7.1.10 Syringe filter
- 7.1.11 Balance capable of weighing to ± 0.1 mg
- 7.1.12 HPLC System capable of injecting 10 µL and equipped with a UV detector capable of operating at 254 nm.

7.2 SOLUTION PREPARATION
- 7.2.1 **Mobile Phase**: Dissolve 4.0 g of chloroacetic acid in 400 mL of water, adjust with ammonium hydroxide to a pH of 3.0 if necessary, add 600 mL of acetonitrile, and mix.

7.2.2 **Sensitivity Solution:** 0.005 mg/mL of USP Ibuprofen RS in Mobile phase

7.2.3 **System Suitability Solution:** 10.0 mg/mL of USP Ibuprofen RS and 0.01 mg/mL each of USP Ibuprofen Related Compound C RS and USP Ibuprofen Related Compound J RS in Mobile phase

7.2.4 **Standard Solution**: 0.02 mg/mL of USP Ibuprofen RS and 0.01 mg/mL each of USP Ibuprofen Related Compound C RS and USP Ibuprofen Related Compound J RS in Mobile phase

7.2.5 **Sample Solution**: Nominally 10.0 mg/mL of ibuprofen prepared as follows. Transfer NLT 10 Tablets to a suitable volumetric flask and add about 50% final volume of Mobile phase. Shake on a mechanical shaker for at least 60 min or until the Tablets are disintegrated. Dilute with Mobile phase to volume. Centrifuge a portion of the solution at about 3000 rpm for about 10 min or until a clear supernatant is obtained. Use the supernatant for analysis.

7.3 CHROMATOGRAPHIC CONDITIONS

7.3.1 **Column**: XBridge C18, 2.1-mm × 10-cm; 2.5-µm or equivalent

7.3.2 **Flow Rate**: 1.0 mL/min

7.3.3 **Detector**: 254 nm

7.3.4 **Injection Volume**: 10 µL

7.4 SYSTEM SUITABILITY

7.4.1 **Samples**: Sensitivity Solution, System Suitability Solution, and Standard Solution

7.4.2 **Suitability Requirements**:

7.4.2.1 **Resolution**: NLT 2.5 between ibuprofen related compound J and ibuprofen; NLT 2.5 between ibuprofen and ibuprofen related compound C, System Suitability Solution

7.4.2.2 **Signal-to-noise ratio**: NLT 10, Sensitivity Solution

7.4.2.3 **Relative Standard Deviation**: NMT 6.0% for ibuprofen related compound J, ibuprofen, and ibuprofen related compound C, Standard solution

7.5 ANALYSIS

7.5.1 **Samples**: Standard Solution and Sample Solution

7.5.2 **Calculations**:

7.5.2.1 Calculate the percentage of ibuprofen related compound J and ibuprofen related compound C in the portion of Tablets taken:

$$\text{Result} = (r_U/r_S) \times (C_S/C_U) \times 100$$

$r_U$ = peak response of ibuprofen related compound J or ibuprofen related compound C from the Sample solution

$r_S$ = peak response of ibuprofen related compound J or ibuprofen related compound C from the Standard solution

$C_S$ = concentration of USP Ibuprofen Related Compound J RS or USP Ibuprofen Related Compound C RS in the Standard solution (mg/mL)

$C_U$ = nominal concentration of ibuprofen in the Sample solution (mg/mL)

7.5.2.2 Calculate the percentage of any unspecified degradation product in the portion of Tablets taken

$$\text{Result} = (r_U/r_S) \times (C_S/C_U) \times 100$$

$r_U$ = peak response of any individual unspecified degradation product from the Sample solution

$r_S$ = peak response of ibuprofen from the Standard solution

$C_S$= concentration of USP Ibuprofen RS in the Standard solution (mg/mL)

$C_U$ = nominal concentration of ibuprofen in the Sample solution (mg/mL)

7.6 ACCEPTANCE CRITERIA: See Table 1. Disregard any peaks less than 0.05%.

Table 1

| Name | Relative Retention Time | Acceptance Criteria NMT (%) |
|---|---|---|
| Ibuprofen related compound J | 0.47 | 0.2 |
| Ibuprofen | 1.00 | - |
| Ibuprofen related compound C | 1.62 | 0.25 |
| Any unspecified degradation product | - | 0.2 |
| Total degradation products | - | 1.5 |

## 2. Scope

2.1 This method describes a procedure for dissolution testing of Ibuprofen Tablets, 400 mg. This method is adapted from and conforms to all procedures, tests and criteria described in the Dissolution Test section of the monograph for Ibuprofen Tablets in the USP41/NF16.

## 4. References

4.1 SOP-001 Quality Assurance Responsibilities
4.2 SOP-002 Document Control System
4.3 SOP-003 Good Documentation Practices
4.4 SOP-004 Records Management\
4.5 SOP-005 Nonconformances and Investigations
4.6 SOP-006 GMP Training Program
4.7 SOP-013 Material Specifications
4.8 SOP-016 Equipment Qualification
4.9 SOP-020 Equipment Calibration
4.10 SOP-024 Weighing on a Balance
4.11 Current USP/NF

## 7. Procedure

7.1 EQUIPMENT AND REAGENTS
- 7.1.1 Dissolution Apparatus 2 – Hanson Vision Elite 8 Dissolution Tester or equivalent
- 7.1.2 UV/Vis Spectrophotometer
- 7.1.3 Ibuprofen Reference Standard
- 7.1.4 Potassium Phosphate Monobasic, ACS Grade or equivalent
- 7.1.5 Potassium Phosphate Dibasic, ACS Grade or equivalent
- 7.1.6 Hydrochloric Acid, ACS Grade or equivalent
- 7.1.7 Sodium Hydroxide, ACS Grade or equivalent
- 7.1.8 Purified Water
- 7.1.9 Class A Glassware
- 7.1.10 Syringe filter
- 7.1.11 Balance capable of weighing to ± 0.1 mg

7.2 SOLUTION PREPARATION

7.2.1 **Medium**: pH 7.2 Phosphate Buffer
- 7.2.1.1 Prepare approximately 5.8 L of distilled water in a suitable container.
- 7.2.1.2 Add 34.8 g of Potassium phosphate dibasic to the solution.
- 7.2.1.3 Add 13.6 g of Potassium phosphate monobasic to the solution.
- 7.2.1.4 Adjust solution to final desired pH using HCl or NaOH
- 7.2.1.5 Add distilled water until volume is 6 L.

7.2.2 **Standard Solution**:

7.2.2.1 Accurately weigh approximately 44.4 mg of Ibuprofen RS into a 100 mL volumetric flask.

7.2.2.2 Add approximately 90 mL of Medium to the flask and mix thoroughly until completely dissolved. Qs to the mark with Medium.

7.2.3 **Sample Solution**: A filtered portion of the solution under test, suitably diluted with *Medium* to obtain a concentration similar to that of the *Standard solution*

7.3 DISSOLUTION CONDITIONS

7.3.1 **Medium**: pH 7.2 Phosphate Buffer, 900 mL

7.3.2 **Apparatus**: 2 (Paddles)

7.3.3 **Rotation Speed**: 50 rpm

7.3.4 **Time:** 60 min

7.4 INSTRUMENT CONDITIONS

7.4.1 **Wavelength**: 221 nm

7.5 ANALYSIS

7.5.1 **Samples**: Standard solution and Sample solution

7.5.2 **Calculations**: Calculate the percentage of the labeled amount of Ibuprofen ($C_{13}H_{18}O_2$) dissolved:

$$\text{Result} = (A_U/A_S) \times (C_S/C_U) \times D \times 100$$

$A_U$ = absorbance of the Sample solution

$A_S$ = absorbance of the Standard solution

$C_S$ = concentration of USP Ibuprofen RS in the Standard solution (mg/mL)

$C_U$ = nominal concentration of Ibuprofen in the Sample solution (mg/mL)

D = dilution factor for the Sample solution

7.6 ACCEPTANCE CRITERIA: NLT 80% (Q) of the labeled amount of Ibuprofen ($C_{13}H_{18}O_2$) is dissolved

Test Method TMD-017

## 2. Scope

2.1 This method describes a procedure for determination of impurities in Acetaminophen Tablets, 500 mg. This method is adapted from and conforms to all procedures, tests and criteria described in the Organic Impurities section of the monograph for Acetaminophen Tablets in the USP41/NF16.

## 4. References

4.1 SOP-001 Quality Assurance Responsibilities
4.2 SOP-002 Document Control System
4.3 SOP-003 Good Documentation Practices
4.4 SOP-004 Records Management\
4.5 SOP-005 Nonconformances and Investigations
4.6 SOP-006 GMP Training Program
4.7 SOP-013 Material Specifications
4.8 SOP-016 Equipment Qualification
4.9 SOP-020 Equipment Calibration
4.10 SOP-024 Weighing on a Balance
4.11 Current USP/NF

## 7. Procedure

7.1 EQUIPMENT AND REAGENTS

7.1.1 Acetaminophen Reference Standard
7.1.2 4-Aminophenol Reference Standard
7.1.3 Ammonium Formate, ACS Grade or equivalent
7.1.4 Formic Acid, ACS Grade or equivalent
7.1.5 Ammonium Acetate, ACS Grade or equivalent
7.1.6 Trifluoroacetic Acid, ACS Grade or equivalent
7.1.7 Water, HPLC Grade or equivalent
7.1.8 Acetonitrile, HPLC Grade
7.1.9 Methanol, HPLC Grade
7.1.10 Class A Glassware
7.1.11 Syringe filter
7.1.12 Balance capable of weighing to ± 0.1 mg
7.1.13 Gradient HPLC System capable of injecting 25 µL, maintaining column temperature at 40 °C, and equipped with a UV detector capable of operating at 272 nm.

7.2 SOLUTION PREPARATION

7.2.1 **Buffer:** Dissolve 1.9 g of Ammonium Formate in 1 L of Water. Add 1.0 mL of Formic Acid.

7.2.2 **Solution A**: Dissolve 3.1 g of Ammonium Acetate in 1 L of Water. Add 1.0 mL of Trifluoroacetic Acid.

7.2.3 **Solution B**: Acetonitrile, Methanol, and Water (10:75:15)

**7.2.4** **Solution C:** Dissolve 3.1 g of ammonium acetate in 1000 mL of Solution B. Add 1.0 mL of Trifluoroacetic acid.

**7.2.5** **Mobile Phase**: See Table 1. Return to original conditions and re-equilibrate the system for 4 min.

Table 1

| **Time (min)** | **Solution A (%)** | **Solution C (%)** |
|---|---|---|
| **0** | **97** | **3** |
| **5** | **70** | **30** |
| **10** | **10** | **90** |
| **11** | **10** | **90** |
| **11.1** | **97** | **3** |
| **15** | **97** | **3** |

7.2.6 **Diluent**: Methanol and Buffer (5:95)

7.2.7 **Sensitivity Solution:** 0.000175 mg/mL of USP 4-Aminophenol RS in Diluent. Sonicate to dissolve, if necessary.

7.2.8 **Standard Solution**: 0.00175 mg/mL of USP 4-Aminophenol RS and 0.0035 mg/mL of USP Acetaminophen RS in Diluent. Sonicate to dissolve, if necessary.

7.2.9 **Sample Stock Solution**: Nominally 5 mg/mL of Acetaminophen in Diluent from NLT 10 Tablets. [NOTE—It is recommended to shake on a flat bed at low speed (180 oscillations/min) to dissolve, if necessary.]

7.2.10 **Sample solution**: Nominally 3.5 mg/mL of Acetaminophen in Diluent prepared as follows. Pass a portion of the Sample stock solution through a suitable filter of 0.2-µm pore size. Discard the first 2 mL of the filtrate. Dilute a suitable volume of the filtrate with Diluent to volume.

7.3 CHROMATOGRAPHIC CONDITIONS

7.3.1 **Column**: XBridge C18, 4.6-mm × 15-cm; 3-µm packing or equivalent

7.3.2 **Flow Rate**: 0.9 mL/min

7.3.3 **Detector**: 272 nm

7.3.4 **Injection Volume**: 25 µL

7.3.5 **Column Temperature**: 40 °C

7.4 SYSTEM SUITABILITY

7.4.1 **Sample**: Sensitivity Solution and Standard Solution

7.4.2 **Suitability Requirements**:

7.4.2.1 **Relative Standard Deviation**: NMT 5.0% for 4-Aminophenol and Acetaminophen, Standard solution

7.4.2.2 **Signal-to-noise ratio**: NLT 10 for 4-Aminophenol, Sensitivity solution

7.5 ANALYSIS

7.5.1 **Samples**: Standard Solution and Sample Solution

7.5.2 **Calculations**:

7.5.2.1 Calculate the percentage of 4-aminophenol in the portion of Tablets taken:

$$\text{Result} = (r_U/r_S) \times (C_S/C_U) \times 100$$

$r_U$ = peak response of 4-aminophenol from the Sample solution
$r_S$ = peak response of 4-aminophenol from the Standard solution
$C_S$ = concentration of USP 4-Aminophenol RS in the Standard solution (mg/mL)
$C_U$ = nominal concentration of acetaminophen in the Sample solution (mg/mL)

7.5.2.2 Calculate the percentage of any unspecified impurity in the portion of Tablets taken:

$$\text{Result} = (r_U/r_S) \times (C_S/C_U) \times 100$$

$r_U$ = peak response of any unspecified impurity from the Sample solution
$r_S$ = peak response of acetaminophen from the Standard solution
$C_S$ = concentration of USP Acetaminophen RS in the Standard solution (mg/mL)
$C_U$ = nominal concentration of acetaminophen in the Sample solution (mg/mL)

7.6 ACCEPTANCE CRITERIA: See Table 2.

Table 2

| Name | Relative Retention Time | Acceptance Criteria NMT (%) |
|---|---|---|
| 4-Aminophenol | 0.53 | 0.15 |
| Acetaminophen | 1.0 | - |
| Any unspecified impurity | | 0.15 |
| Total impurities | | 0.60 |

## 2. Scope

2.1 This method describes a procedure for dissolution testing of Acetaminophen Tablets, 500 mg. This method is adapted from and conforms to all procedures, tests and criteria described in the Dissolution Test section of the monograph for Acetaminophen Tablets in the USP41/NF16.

## 4. References

4.1 SOP-001 Quality Assurance Responsibilities
4.2 SOP-002 Document Control System
4.3 SOP-003 Good Documentation Practices
4.4 SOP-004 Records Management\
4.5 SOP-005 Nonconformances and Investigations
4.6 SOP-006 GMP Training Program
4.7 SOP-013 Material Specifications
4.8 SOP-016 Equipment Qualification
4.9 SOP-020 Equipment Calibration
4.10 SOP-024 Weighing on a Balance
4.11 Current USP/NF

## 7. Procedure

7.1 EQUIPMENT AND REAGENTS
7.1.1 Dissolution Apparatus 2 – Hanson Vision Elite 8 Dissolution Tester or equivalent
7.1.2 UV/Vis Spectrophotometer
7.1.3 Acetaminophen Reference Standard
7.1.4 Potassium Phosphate Monobasic, ACS Grade or equivalent
7.1.5 Potassium Phosphate Dibasic, ACS Grade or equivalent
7.1.6 Hydrochloric Acid, ACS Grade or equivalent
7.1.7 Sodium Hydroxide, ACS Grade or equivalent
7.1.8 Purified Water
7.1.9 Class A Glassware
7.1.10 Syringe filter
7.1.11 Balance capable of weighing to ± 0.1 mg

7.2 SOLUTION PREPARATION
7.2.1 **Medium**: pH 5.8 Phosphate Buffer
7.2.1.1 Prepare approximately 5.8 L of distilled water in a suitable container.
7.2.1.2 Add 2.51 g of Potassium phosphate dibasic to the solution.
7.2.1.3 Add 38.9 g of Potassium phosphate monobasic to the solution.
7.2.1.4 Adjust solution to final desired pH using HCl or NaOH
7.2.1.5 Add distilled water until volume is 6 L.

7.2.2 **Standard Solution**:

7.2.2.1 Accurately weigh approximately 55.5 mg of Acetaminophen RS into a 100 mL volumetric flask.

7.2.2.2 Add approximately 90 mL of Medium to the flask and mix thoroughly until completely dissolved. Qs to the mark with Medium.

7.2.3 **Sample Solution**: A filtered portion of the solution under test, suitably diluted with *Medium* to obtain a concentration similar to that of the *Standard solution*

7.3 DISSOLUTION CONDITIONS

7.3.1 **Medium**: pH 5.8 Phosphate Buffer, 900 mL

7.3.2 **Apparatus**: 2 (Paddles)

7.3.3 **Rotation Speed**: 50 rpm

7.3.4 **Time:** 30 min

7.4 INSTRUMENT CONDITIONS

7.4.1 **Wavelength**: 243 nm

7.5 ANALYSIS

7.5.1 **Samples**: Standard solution and Sample solution

7.5.2 **Calculations**: Calculate the percentage of the labeled amount of acetaminophen ($C_8H_9NO_2$) dissolved.

$$\text{Result} = (A_U/A_S) \times (C_S/C_U) \times D \times 100$$

| | | |
|---|---|---|
| $A_U$ | = | absorbance of the Sample solution |
| $A_S$ | = | absorbance of the Standard solution |
| $C_S$ | = | concentration of USP Acetaminophen RS in the Standard solution (mg/mL) |
| $C_U$ | = | nominal concentration of Ibuprofen hydrochloride in the Sample solution (mg/mL) |
| D | = | dilution factor for the Sample solution |

7.6 ACCEPTANCE CRITERIA: NLT 80% (Q) of the labeled amount of acetaminophen ($C_8H_9NO_2$) is dissolved

## 2. Scope

2.1 This method describes a procedure for determination of impurities in Promethazine Hydrochloride Tablets, 25 mg. This method is adapted from and conforms to all procedures, tests and criteria described in the Organic Impurities section of the monograph for Promethazine Hydrochloride Tablets in the USP41/NF16.

## 4. References

4.1 SOP-001 Quality Assurance Responsibilities
4.2 SOP-002 Document Control System
4.3 SOP-003 Good Documentation Practices
4.4 SOP-004 Records Management\
4.5 SOP-005 Nonconformances and Investigations
4.6 SOP-006 GMP Training Program
4.7 SOP-013 Material Specifications
4.8 SOP-016 Equipment Qualification
4.9 SOP-020 Equipment Calibration
4. SOP-024 Weighing on a Balance
4. Current USP/NF

## 7. Procedure

7.1 EQUIPMENT AND REAGENTS

7.1.1 Promethazine Hydrochloride Reference Standard
7.1.2 Promethazine Related Compound B Reference Standard
7.1.3 Triethylamine, ACS Grade or equivalent
7.1.4 Water, HPLC Grade or equivalent
7.1.5 Ammonium Acetate, ACS Grade or equivalent
7.1.6 Methanol, HPLC Grade or equivalent
7.1.7 Acetonitrile, HPLC Grade or equivalent
7.1.8 Class A Glassware
7.1.9 Syringe filter, 0.45 μm
7.1.10 Balance capable of weighing to ± 0.1 mg
7.1.11 Gradient HPLC System capable of injecting 15 μL and equipped with a UV detector capable of operating at 234 and 249 nm.

7.2 SOLUTION PREPARATION

7.2.1 **Diluent:** Methanol and Triethylamine (999:1).

7.2.2 **Buffer:** 3.7 g/L of Ammonium Acetate in water.

7.2.3 **Solution A:** Buffer and Acetonitrile (700:300)

7.2.4 **Solution B:** Acetonitrile

7.2.5 **Mobile Phase:**

Table 1

| Time (min) | Solution A (%) | Solution B (%) |
|---|---|---|
| 0 | 100 | 0 |
| 10 | 60 | 40 |
| 18 | 60 | 40 |
| 18.1 | 100 | 0 |
| 25 | 100 | 0 |

7.2.6 **System Suitability Stock Solution:** 0.5 mg/mL of USP Promethazine Related Compound B RS in Diluent.

7.2.7 **Standard Stock Solution**: 0.5 mg/mL of USP Promethazine Hydrochloride RS in Diluent.

7.2.8 **System Suitability Solution**: 5 µg/mL each of USP Promethazine Hydrochloride RS and USP Promethazine Related Compound B RS from the Standard Stock solution and System Suitability Stock Solution, respectively

7.2.9 **Standard Solution**: 5 µg/mL of USP Promethazine Hydrochloride RS from the Standard Stock Solution

7.2.10 **Sensitivity Solution:** 0.25 µg/mL of USP Promethazine Hydrochloride RS from the Standard Solution

7.2.11 **Sample Solution**: Nominally 0.5 mg/mL of promethazine hydrochloride from powdered Tablets (NLT 20) prepared as follows. Transfer a quantity of powdered Tablets, equivalent to 50 mg of promethazine hydrochloride, to a volumetric flask of appropriate size and add 75% of the flask volume of Diluent. Shake the flask for NLT 5 min and dilute with Diluent to volume. Pass a portion through a suitable filter.

7.3 CHROMATOGRAPHIC CONDITIONS

7.3.1 **Column**: XBridge C184.6-mm × 15-cm; 5-µm or equivalent

7.3.2 **Column Temperature:** 30°C

7.3.3 **Flow Rate**: 1.4 mL/min

7.3.4 **Detector**: 234 and 249 nm

7.3.5 **Injection Volume**: 15 µL

7.4 SYSTEM SUITABILITY

7.4.1 **Samples**: System Suitability Solution, Standard Solution, and Sensitivity Solution [NOTE—See Table 2 for the relative retention times.]

7.4.2 **Suitability Requirements**:

7.4.2.1 **Resolution:** NLT 5.0 between Promethazine and Promethazine Related Compound B, System Suitability Solution

7.4.2.2 **Relative Standard Deviation:** NMT 3.0% at 234 and 249 nm, Standard Solution

7.4.2.3 **Signal-to-noise ratio**: NLT 10 at 234 and 249 nm, Sensitivity solution

7.5 ANALYSIS

7.5.1 **Samples**: Standard Solution and Sample Solution

7.5.2 **Calculations**:

7.5.2.1 Calculate the percentage of Promethazine Sulfoxide in the portion of Tablets taken:

$$Result = (r_U/r_S) \times (C_S/C_U) \times (1/F) \times 100$$

$r_U$ = peak response of promethazine sulfoxide at 234 nm from the Sample Solution

$r_S$= peak response of promethazine hydrochloride at 234 nm from the Standard Solution

$C_S$ = concentration of USP Promethazine Hydrochloride RS in the Standard Solution (mg/mL)

$C_U$ = nominal concentration of promethazine hydrochloride in the Sample Solution (mg/mL)

F = relative response factor (see Table 2)

7.5.2.2 Calculate the percentage of all other degradation products in the portion of Tablets taken:

$$Result = (r_U/r_S) \times (C_S/C_U) \times (1/F) \times 100$$

$r_U$ = peak response of each degradation product at 249 nm from the Sample solution

$r_S$ = peak response of promethazine hydrochloride at 249 nm from the Standard solution

$C_S$ = concentration of USP Promethazine Hydrochloride RS in the Standard solution (mg/mL)

$C_U$ = nominal concentration of promethazine hydrochloride in the Sample solution (mg/mL)

F = relative response factor (see Table 2)

7.6 ACCEPTANCE CRITERIA: See Table 2. Disregard peaks that are less than 0.05%.

Table 2

| Name | Relative Retention Time | Relative Response Factor | Acceptance Criteria NMT (%) |
|---|---|---|---|
| Promethazine sulfoxide[a] | 0.28 | 2.1 | 0.5 |
| Desmethyl promethazine[b] | 0.71 | 1.0 | 0.5 |
| Promethazine | 1.0 | - | - |
| Promethazine related compound B[c] | 1.3 | - | - |
| Phenothiazine | 1.7 | 2.0 | 0.5 |
| Any individual unspecified degradation product | - | 1.0 | 0.2 |
| Total degradation products | - | - | 1.0 |

[a] *N*,*N*-Dimethyl-1-(10*H*-phenothiazin-10-yl)propan-2-amine sulfoxide.

[b] *N*-Methyl-1-(10*H*-phenothiazin-10-yl)propan-2-amine.

[c] This is a process impurity and is included for identification only. It is not to be reported and not to be included in the total degradation products.

## 2. Scope

2.1 This method describes a procedure for dissolution testing of Promethazine Hydrochloride Tablets, 25 mg. This method is adapted from and conforms to all procedures, tests and criteria described for Test 1 in the Dissolution Test section of the monograph for Promethazine Hydrochloride Tablets in the USP41/NF16.

## 4. References

4. SOP-001 Quality Assurance Responsibilities
4. SOP-002 Document Control System
4. SOP-003 Good Documentation Practices
4. SOP-004 Records Management\
4. SOP-005 Nonconformances and Investigations
4. SOP-006 GMP Training Program
4. SOP-013 Material Specifications
4. SOP-016 Equipment Qualification
4. SOP-020 Equipment Calibration
4.10 SOP-024 Weighing on a Balance
4.11 Current USP/NF

## 7. Procedure

7.1 EQUIPMENT AND REAGENTS

7.1.1 Dissolution Apparatus 1 – Hanson Vision Elite 8 Dissolution Tester or equivalent
7.1.2 UV/Vis Spectrophotometer
7.1.3 Promethazine Hydrochloride Reference Standard
7.1.4 0.01 N Hydrochloric Acid, ACS Grade or equivalent
7.1.5 Class A Glassware
7.1.6 Syringe filter, 0.45 µm
7.1.7 Balance capable of weighing to ± 0.1 mg

7.2 SOLUTION PREPARATION

7.2.1 **Medium**: 0.01 N Hydrochloric Acid

7.2.2 **Standard Stock Solution**:

7.2.2.1 Accurately weigh approximately 27.8 mg of Promethazine Hydrochloride RS into a 100 mL volumetric flask.

7.2.2.2 Add approximately 90 mL of Medium to the flask and mix thoroughly until completely dissolved. Qs to the mark with Medium.

7.2.3 **Standard Solution**:

7.2.3.1 Accurately pipet 10 mL of the Standard Stock Solution into a 100 mL volumetric flask.

7.2.3.2 Qs to the mark with Medium and mix thoroughly.

7.2.4 **Sample Solution**: A filtered portion of the solution under test, suitably dilutedwith Medium to obtain a concentration similar to that of the Standard Solution

7.3 DISSOLUTION CONDITIONS

7.3.1 **Medium**: 0.01 N Hydrochloric Acid, 900 mL

7.3.2 **Apparatus**: 1 (Paddles)

7.3.3 **Rotation Speed**: 100 rpm

7.3.4 **Time:** 45 min

7.4 INSTRUMENT CONDITIONS

7.4.1 **Wavelength**: 249 nm

7.5 ANALYSIS

7.5.1 **Samples**: Standard Solution and Sample Solution

7.5.2 **Calculations**: Calculate the percentage of the labeled amount of Promethazine Hydrochloride ($C_{17}H_{20}N_2S \cdot HCl$) dissolved:

$$\text{Result} = (A_U/A_S) \times (C_S/C_U) \times D \times 100$$

| | | |
|---|---|---|
| $A_U$ | = | absorbance of the *Sample solution* |
| $A_S$ | = | absorbance of the *Standard solution* |
| $C_S$ | = | concentration of USP [redacted] Hydrochloride RS in the *Standard solution* (mg /mL) |
| $C_U$ | = | nominal concentration of romethazine hydrochloride in the *Sample solution* (mg/mL) |
| $D$ | = | dilution factor for the *Sample solution* |

7.6 ACCEPTANCE CRITERIA: NLT 75% (Q) of the labeled amount of Promethazine Hydrochloride ($C_{17}H_{20}N_2S \cdot HCl$) is dissolved

## 2. Scope

2.1 This method describes a procedure for determination of Impurities in Amoxicillin Capsules, 500 mg. This method is adapted from and conforms to all procedures, tests and criteria described in the Organic Impurities section of the monograph for Amoxicillin Capsules in the USP41/NF16.

## 4. References

4.1 SOP-001 Quality Assurance Responsibilities
4.2 SOP-002 Document Control System
4.3 SOP-003 Good Documentation Practices
4.4 SOP-004 Records Management\
4.5 SOP-005 Nonconformances and Investigations
4.6 SOP-006 GMP Training Program
4.7 SOP-013 Material Specifications
4.8 SOP-016 Equipment Qualification
4.9 SOP-020 Equipment Calibration
4.10 SOP-024 Weighing on a Balance
4. Current USP/NF

## 7. Procedure

7.1 EQUIPMENT AND REAGENTS

7.1.1 Amoxicillin Reference Standard
7.1.2 Amoxicillin Related Compound C Reference Standard
7.1.3 Amoxicillin Related Compound H Reference Standard
7.1.4 Monobasic Potassium Phosphate, ACS Grade or equivalent
7.1.5 20% Sodium Hydroxide Solution
7.1.6 Water, HPLC Grade or equivalent
7.1.7 Acetonitrile, HPLC Grade
7.1.8 Class A Glassware
7.1.9 Syringe filter
7.1.10 Balance capable of weighing to ± 0.1 mg
7.1.11 HPLC System capable of injecting 10 µL and equipped with a UV detector capable of operating at 230 nm.

7.2 SOLUTION PREPARATION

7.2.1 **Solution A**: Dissolve 6.8 g/L of monobasic potassium phosphate in water. Adjust with a 20% (w/v) solution of sodium hydroxide to a pH of 5.0 ± 0.1.
7.2.2 **Solution B**: Acetonitrile

**7.2.3 Mobile Phase**: See Table 1.

Table 1

| Time (min) | Solution A (%) | Solution B (%) |
|---|---|---|
| 0 | 100 | 0 |
| 5 | 100 | 0 |
| 25 | 94 | 6 |
| 40 | 84 | 16 |
| 50 | 84 | 16 |
| 51 | 100 | 0 |
| 60 | 100 | 0 |

7.2.4 **Impurity Stock Solution:** 0.15 mg/mL each of USP Amoxicillin Related Compound C RS and USP Amoxicillin Related Compound H RS in Solution A, prepared as follows. Transfer a weighed amount of USP Amoxicillin Related Compound C RS and USP Amoxicillin Related Compound H RS to a suitable volumetric flask. Add acetonitrile to fill 10% of the flask volume and Solution A to fill 60% of the flask volume. Sonicate to dissolve and dilute with Solution A to volume.

7.2.5 **System Suitability Solution:** 1.5 mg/mL of USP Amoxicillin RS and 0.015 mg/mL each of USP Amoxicillin Related Compound C RS and USP Amoxicillin Related Compound H RS in Solution A prepared as follows. Transfer a weighed amount of USP Amoxicillin RS to a suitable volumetric flask. Add Solution A to fill 60% of the flask volume. Add an appropriate volume of Impurity stock solution to the volumetric flask. Sonicate to dissolve and dilute with Solution A to volume.

7.2.6 **Standard Solution**: 0.017 mg/mL of USP Amoxicillin RS in Solution A. Sonicate if necessary to dissolve. Use this solution immediately after preparation.

7.2.7 **Sample solution**: Nominally 1.5 mg/mL of amoxicillin in Solution A from the Capsules, prepared as follows. Transfer Capsule powder equivalent to 75 mg of amoxicillin into a 50-mL volumetric flask. Add Solution A to fill 60% of the final flask volume. Sonicate for 15 min and dilute with Solution A to volume. Pass through a suitable filter of 0.45-µm pore size. Use this solution immediately after preparation.

7.3 CHROMATOGRAPHIC CONDITIONS

7.3.1 **Column**: XBridge C8, 4.6-mm × 15-cm; 5-µm packing packing or equivalent

7.3.2 **Column Temperature:** 40 ºC

7.3.3 **Flow Rate**: 2 mL/min

7.3.4 **Detector**: 230 nm

7.3.5 **Injection Volume**: 20 µL

7.4 SYSTEM SUITABILITY

7.4.1 **Sample**: System Suitability Solution and Standard Solution. [NOTE—See Table 2 for relative retention times.]

7.4.2 **Suitability Requirements**:

**7.4.2.1 Resolution:** NLT 1.5 between amoxicillin related compound C and amoxicillin related compound H, System suitability solution

**7.4.2.2 Relative Standard Deviation**: NMT 5.0%, Standard Solution.

7.5 ANALYSIS

7.5.1 **Samples**: Standard solution and Sample solution

7.5.2 **Calculations**:

Calculate the percentage of each degradation product in the portion of Capsules taken:

$$\text{Result} = (r_U/r_S) \times (C_S/C_U) \times P \times (F_1/F_2) \times 100$$

$r_U$ = peak response of each degradation product from the Sample solution

$r_S$ = peak response of amoxicillin from the Standard solution

$C_S$ = concentration of USP Amoxicillin RS in the Standard solution (mg/mL)

$C_U$ = nominal concentration of amoxicillin in the Sample solution (mg/mL)

P = potency of amoxicillin in USP Amoxicillin RS (µg/mg)

$F_1$ = conversion factor, 0.001 mg/µg

$F_2$ = relative response factor (see Table 2)

7.6 ACCEPTANCE CRITERIA: See Table 2. Disregard any peak less than 0.05%.

Table 2

| Name | Relative Retention Time | Relative Response Factor | Acceptance Criteria NMT (%) |
|---|---|---|---|
| Amoxicillin related compound I [a,b] (D-hydroxyphenylglycine) | 0.19 | — | — |
| Amoxicillin related compound D [c,d] (amoxicillin open ring) | 0.36, 0.47 | 0.8 | 2.4 |
| Amoxicillin related compound A [a,e] (6-aminopenicillanic acid) | 0.66 | — | — |
| Amoxicillin related compound B [a,f] (L-amoxicillin) | 0.82 | — | — |
| Amoxicillin | 1.0 | 1.0 | — |
| Amoxicillin related compound E [d,g] | 2.5, 3.32 | 1.0 | 3.6 |
| Amoxicillin related compound G [a,h] (D-hydroxyphenylglycylamoxicillin) | 3.03 | — | — |
| Amoxicillin related compound C [i] (amoxicillin rearrangement product) | 3.63, 3.84 | 0.98 | 2.0 |
| Amoxicillin related compound H [a,j] (N-pivaloyl pHPG) | 4.03 | — | — |
| Amoxicillin related compound F [k] (pyrazine-2-ol) | 4.12 | 1.1 | 1.0 |
| Amoxicillin related compound K [d,l] (amoxicilloic acid dimers 1 and 2) | 4.39, 4.75 | 0.64 | 1.0 |
| 6-APA amoxicillin amide [a,m] | 6.24 | — | — |
| Amoxicilloic amoxilloic acid dimers 1, 2, 3, and 4 [d] | 6.18, 6.40, 6.56 | 0.46 | 1.0 |
| Amoxicillin related compound J [n] (amoxicillin open ring dimer) | 7.02 | 0.64 | 2.0 |
| N-Pivaloyl amoxicillin | 7.96 | — | — |
| Any individual unspecified degradation product | - | — | 1.0 |
| Total impurities | - | — | 7.0 |

[a] These are process impurities that are controlled in the drug substance. They are listed here for reference only and are not to be reported.

[b] (*R*)-2-Amino-2-(4-hydroxyphenyl)acetic acid.

[c] (4*S*)-2-{[(*R*)-2-Amino-2-(4-hydroxyphenyl)acetamido](carboxy)methyl}-5,5-dimethylthiazolidine-4-carboxylic acid.

[d] Some chromatographic systems may resolve the peaks from isomers, and the limit is for the sum of all the isomers.
[e] (2*S*,5*R*,6*R*)-6-Amino-3,3-dimethyl-7-oxo-4-thia-1-azabicyclo[3.2.0]heptane-2-carboxylic acid.
[f] (2*S*,5*R*,6*R*)-6-[(*S*)-2-Amino-2-(4-hydroxyphenyl)acetamido]-3,3-dimethyl-7-oxo-4-thia-1-azabicyclo[3.2.0]heptane-2-carboxylic acid.
[g] (4*S*)-2-{[(*R*)-2-Amino-2-(4-hydroxyphenyl)acetamido]methyl}-5,5-dimethylthiazolidine-4-carboxylic acid and (4*R*)-2-{[(*S*)-2-amino-2-(4-hydroxyphenyl)acetamido]methyl}-5,5-dimethylthiazolidine-4-carboxylic acid.
[h] (2*S*,5*R*,6*R*)-6-{(*R*)-2-Amino-2-(4-hydroxyphenyl)acetamido]-2-(4-hydroxyphenyl)acetamido}-3,3-dimethyl-7-oxo-4-thia-1-azabicyclo[3.2.0]heptane-2-carboxylic acid.
[i] (4*S*)-2-[5-(4-Hydroxyphenyl)-3,6-dioxopiperazin-2-yl]-5,5-dimethylthiazolidine-4-carboxylic acid.
[j] (*R*)-2-(4-Hydroxyphenyl)-2-pivalamidoacetic acid.
[k] 3-(4-Hydroxyphenyl) pyrazin-2-ol.
[l] Oligomers of penicilloic acids of amoxicillin.
[m] (2*S*,5*R*,6*R*)-6-[[(2*S*,5*R*,6*R*)-6-[(2*R*)-2-Amino-2-(4-hydroxyphenyl)acetamido]-3,3-dimethyl-7-oxo-4-thia-1-azabicyclo[3.2.0]heptane-2-carbonyl]amino]-3,3-dimethyl-7-oxo-4-thia-1-azabicyclo[3.2.0]heptane-2-carboxylic acid.
[n] Co-oligomers of amoxicillin and penicilloic acids of amoxicillin.

Test Method TMD-024

## 2. Scope

2.1 This method describes a procedure for dissolution testing of Amoxicillin Capsules, 500 mg. This method is adapted from and conforms to all procedures, tests and criteria described in the Dissolution Test section of the monograph for Amoxicillin Capsules in the USP41/NF16.

## 4. References

4.1 SOP-001 Quality Assurance Responsibilities
4.2 SOP-002 Document Control System
4.3 SOP-003 Good Documentation Practices
4.4 SOP-004 Records Management\
4.5 SOP-005 Nonconformances and Investigations
4.6 SOP-006 GMP Training Program
4.7 SOP-013 Material Specifications
4.8 SOP-016 Equipment Qualification
4.9 SOP-020 Equipment Calibration
4.10 SOP-024 Weighing on a Balance
4.11 Current USP/NF

## 7. Procedure

7. EQUIPMENT AND REAGENTS
- 7.1.1 Dissolution Apparatus 2 – Hanson Vision Elite 8 Dissolution Tester or equivalent
- 7.1.2 UV/Vis Spectrophotometer
- 7.1.3 Amoxicillin Reference Standard
- 7.1.4 Purified Water
- 7.1.5 Class A Glassware
- 7.1.6 Syringe filter
- 7.1.7 Balance capable of weighing to ± 0.1 mg

7.2 SOLUTION PREPARATION
- 7.2.1 **Standard Solution**:
  - 7.2.1.1 Accurately weigh approximately 55.5 mg of Amoxicillin RS into a 100 mL volumetric flask.
  - 7.2.1.2 Add approximately 90 mL of Medium to the flask and mix thoroughly until completely dissolved. Qs to the mark with Medium.
- 7.2.2 **Sample Solution**:
  Pass a portion of the solution under test through a suitable filter. Dilute with Medium, if necessary, to a concentration that is similar to that of the Standard solution.

7.3 DISSOLUTION CONDITIONS

7.3.1 **Medium**: Water, 900 mL

7.3.2 **Apparatus**: 2 (Paddles)

7.3.3 **Rotation Speed**: 75 rpm

7.3.4 **Time:** 60 min

7.4 INSTRUMENT CONDITIONS

7.4.1 **Wavelength**: 272 nm

7.5 ANALYSIS

7.5.1 **Samples**: Standard solution and Sample solution

7.5.2 **Calculations**: Calculate the percentage of the labeled amount of Amoxicillin ($C_8H_9NO_2$) dissolved.

$$\text{Result} = (A_U/A_S) \times (C_S/C_U) \times D \times 100$$

7.6 ACCEPTANCE CRITERIA: NLT 80% (Q) of the labeled amount of Amoxicillin ($C_{16}H_{19}N_3O_5S$) is dissolved.

| | | |
|---|---|---|
| $A_U$ | = | absorbance of the Sample solution |
| $A_S$ | = | absorbance of the Standard solution |
| $C_S$ | = | concentration of USP Amoxicillin RS in the Standard solution (mg/mL) |
| $C_U$ | = | nominal concentration of Amoxicillin in the Sample solution (mg/mL) |
| D | = | dilution factor for the Sample solution |

Drug assay HPLC methods and conditions.

1. **METHOD TMD-013: IBUPROFEN 400 MG TABLET**

1.1. EQUIPMENT AND REAGENTS

1.1.1. Ibuprofen Reference Standard

1.1.2. Chloroacetic Acid, ACS Grade or equivalent

1.1.3. Ammonium Hydroxide, ACS Grade or equivalent

1.1.4. Water, HPLC Grade or equivalent

1.1.5. Acetonitrile, HPLC Grade or equivalent

1.1.6. Methanol, HPLC Grade or equivalent

1.1.7. Class A Glassware

1.1.8. Syringe filter

1.1.9. Balance capable of weighing to ± 0.1 mg

1.1.10. HPLC System capable of injecting 10 µL and equipped with a UV detector capable of operating at 254 nm.

1.2. SOLUTION PREPARATION

1.2.1. **Mobile Phase:** Dissolve 4.0 g of chloroacetic acid in 400 ml of water, adjust with ammonium hydroxide to a pH of 3.0 if necessary, add 600 ml of acetonitrile, and mix.

1.2.2. **Standard Solution:** 10.0 mg/ml of USP Ibuprofen RS in Mobile phase.

1.2.3. **Sample solution:** Nominally 10.0 mg/ml of ibuprofen prepared as follows. Transfer NL T 10 Tablets to a suitable volumetric flask and add about 50% final volume of Mobile phase. Shake on a mechanical shaker for at least 60 min or until the Tablets are disintegrated. Dilute with Mobile phase to volume. Centrifuge a portion of the solution at about 3000 rpm for about 1 O min or until a clear supernatant is obtained. Use the supernatant for analysis.

1.3. CHROMATOGRAPHIC CONDITIONS

1.3.1. **Column:** XBridge C18, 2.1-mm x 10-cm; 2.5-µm or equivalent

1.3.2. **Flow Rate:** 1.0 mL/min

1.3.3. **Detector:** 254 nm

1.3.4. **Injection Volume:** 10 µL

1.4. 7.4 SYSTEM SUITABILITY

1.4.1. **Sample:** Standard Solution

1.4.2. **Suitability Requirements:**

1.4.2.1. Tailing Factor NMT 2.5

1.4.2.2. The peak areas five consecutive injections of sample have a %RSD not more than (NMT) 2.0%

1.5. ANALYSIS

1.5.1. **Samples:** Standard solution and Sample solution

1.5.2. **Calculations:** Calculate the percentage of the labeled amount of ibuprofen (C13H18O2) in the portion of Tablets taken:

Result = (ru/rs) x (Cs/Cu) x 100

ru = peak response of ibuprofen from the Sample solution

rs = peak response of ibuprofen from the Standard solution

Cs = concentration of USP Ibuprofen RS in the Standard solution (mg/ml)

Cu= nominal concentration of ibuprofen in the Sample solution (mg/ml)

1.6. ACCEPTANCE CRITERIA: 90.0%-110.0%

## 2. METHOD TMD-016. ACETAMINOPHEN 500 MG TABLETS

2.1. EQUIPMENT AND REAGENTS

2.1.1. Acetaminophen Reference Standard
2.1.2. Glacial Acetic Acid, ACS Grade or equivalent
2.1.3. Water, HPLC Grade or equivalent
2.1.4. Methanol, HPLC Grade
2.1.5. Class A Glassware
2.1.6. Syringe filter
2.1.7. Balance capable of weighing to ± 0.1 mg
2.1.8. Gradient HPLC System capable of injecting 10 µL, maintaining column temperature at 40 °C, and equipped with a UV detector capable of operating at 243 nm.

2.2. 7.2 SOLUTION PREPARATION

2.2.1. Solution A: 1 % v/v Glacial Acetic Acid in water.
2.2.2. Solution B: Methanol
2.2.3. Mobile Phase:

| **Time (min)** | **Solution A** (%) | **Solution B** (%) |
|---|---|---|
| **0.0** | 90 | 10 |
| **4.0** | 90 | 10 |
| **4.1** | 20 | 80 |
| **6.0** | 20 | 80 |
| **6.1** | 90 | 10 |
| **10.0** | 90 | 10 |

2.2.4. **Diluent:** Methanol:Water (10:90)
2.2.5. **Standard Solution:** 0.01 mg/ml of USP Acetaminophen RS in Diluent.
2.2.6. **Sample stock solution:** Nominally 0.1 mg/ml of Acetaminophen in Diluent prepared as follows. Transfer an appropriate amount of acetaminophen from NLT 10 Tablets to a suitable volumetric flask and dilute with Diluent to volume. Centrifuge or pass a portion of this solution through a suitable filter. [NoteSonication or shaking may be necessary.]
2.2.7. **Sample solution:** Nominally 0.01 mg/ml of acetaminophen in Diluent from the Sample stock solution. Pass a portion of this solution through a suitable filter.

2.3. CHROMATOGRAPHIC CONDITIONS

2.3.1. **Column:** XBridge C18, 3.0-mm x 10-cm; 3.5-µm packing or equivalent
2.3.2. **Flow Rate:** 0.5 mL/min
2.3.3. **Detector:** 243 nm
2.3.4. **Injection Volume:** 10 µL
2.3.5. **Column Temperature:** 40 °C

2.4. SYSTEM SUITABILITY

2.4.1. **Sample:** Standard Solution
2.4.2. **Suitability Requirements:**
2.4.2.1. Tailing Factor NMT 2.0
2.4.3. The peak areas five consecutive injections of sample have a %RSD NMT 2.0%

2.5. ANALYSIS

2.5.1. **Samples:** Standard solution and Sample solution
2.5.2. **Calculations:** Calculate the percentage of the labeled amount of ibuprofen (C8H9O2) in the portion of Tablets taken:

Result = (ru/rs) x (Cs/Cu) x 100
ru = peak response of ibuprofen from the Sample solution
rs = peak response of ibuprofen from the Standard solution
Cs = concentration of USP Ibuprofen RS in the Standard solution (mg/ml)
Cu= nominal concentration of ibuprofen in the Sample solution (mg/ml)

2.6. ACCEPTANCE CRITERIA: 90.0%-110.0%

## 3. METHOD TMD-019 PROMETHAZINE HYDROCHLORIDE 25 MG TABLET

3.1. EQUIPMENT AND REAGENTS

3.1.1. Promethazine Hydrochloride Reference Standard
3.1.2. Promethazine Related Compound B Reference Standard
3.1.3. Hydrochloric Acid, ACS Grade or equivalent
3.1.4. Triethylamine, ACS Grade or equivalent
3.1.5. Water, HPLC Grade or equivalent
3.1.6. Acetonitrile, HPLC Grade or equivalent
3.1.7. Class A Glassware
3.1.8. Syringe filter, 0.45 µm
3.1.9. Balance capable of weighing to± 0.1 mg
3.1.10. HPLC System capable of injecting 20 **µL** and equipped with a UV detector
3.1.11. capable of operating at 254 nm.

3.2. SOLUTION PREPARATION

3.2.1. **Diluent:** Dissolve 8.2 ml of Hydrochloric Acid in 1000 ml of water.
3.2.2. **Mobile Phase:** Acetonitrile, Water, and Triethylamine (850:270:1)
3.2.3. **System Suitability Stock Solution:** 1.2 mg/ml of USP Promethazine Related Compound B RS in Diluent. Sonicate to dissolve.
3.2.4. **Standard Solution:** 0.1 mg/ml of USP Promethazine Hydrochloride RS in Diluent. Sonicate to dissolve.
3.2.5. **System Suitability Solution:** 0.09 mg/ml of USP Promethazine Hydrochloride RS and 0.12 mg/ml of USP Promethazine Related Compound B RS in Diluent from the Standard solution and System suitability stock solution, respectively.
3.2.6. **Sample Stock Solution:** Nominally 2.5-5.0 mg/ml of Promethazine Hydrochloride prepared as follows. Transfer 20 Tablets to a volumetric flask of an appropriate size and add 50% of the flask volume of Diluent. Sonicate with swirling for NL T 20 min, or until the Tablets have fully disintegrated. Shake the flask for NL T 15 min and dilute with Diluent to volume.
3.2.7. **Sample solution:** Nominally 0.1 mg/ml of Promethazine Hydrochloride in Diluent from the Sample Stock Solution. Pass a portion through a filter of 0.45 µm pore size and use the clear filtrate.

3.3. CHROMATOGRAPHIC CONDITIONS

3.3.1. **Column:** XBridge C18, 3.9-mm x 30-cm, 10-µm or equivalent
3.3.2. **Flow Rate:** 2.5 ml/min
3.3.3. **Detector:** 254 nm
3.3.4. **Injection Volume:** 20 µl
3.3.5. **Run time:** NLT 2.5 times the retention time of Promethazine

3.4. SYSTEM SUITABILITY

3.4.1. **Samples:** System Suitability Solution and Standard Solution [NOTE-The relative retention times for promethazine related compound B and promethazine are 0.82 and 1.0, respectively.]
3.4.2. **Suitability Requirements:**

3.4.2.1. **Resolution:** NLT 1.5 between promethazine and promethazine related compound B, System Suitability Solution

3.4.2.2. Tailing Factor NMT 1.5

3.4.2.3. The peak areas five consecutive injections of Standard Solution have a %RSD NMT 2.0%

3.5. ANALYSIS

3.5.1. **Samples:** Standard solution and Sample solution

3.5.2. **Calculations:** Calculate the percentage of the labeled amount of ibuprofen (C11H20N2S·HCl) in the portion of Tablets taken:

Result = (ru/rs) x (Cs/Cu) x 100

ru = peak response of ibuprofen from the Sample solution

rs = peak response of ibuprofen from the Standard solution

Cs = concentration of USP Ibuprofen RS in the Standard solution (mg/ml)

Cu= nominal concentration of ibuprofen in the Sample solution (mg/ml)

3.6. ACCEPTANCE CRITERIA: 95.0%-110.0%

## 4. METHOD TMD-022 AMOXICILLIN 500 CAPSULES

4.1. EQUIPMENT AND REAGENTS

4.1.1. Amoxicillin Reference Standard

4.1.2. Monobasic Potassium Phosphate, ACS Grade or equivalent

4.1.3. 45% Potassium Hydroxide Test Solution

4.1.4. Water, HPLC Grade or equivalent

4.1.5. Acetonitrile, HPLC Grade

4.1.6. Class A Glassware

4.1.7. Syringe filter

4.1.8. Balance capable of weighing to ± 0.1 mg

4.1.9. HPLC System capable of injecting 1 O µL and equipped with a UV detector capable of operating at 230 nm.

4.2. SOLUTION PREPARATION

4.2.1. **Buffer:** Dissolve 6.8 g/L of monobasic potassium phosphate in water. Adjust with 45% potassium hydroxide TS to a pH of 5.0 ± 0.1.

4.2.2. **Mobile Phase:** Acetonitrile and Buffer (1 :24)

4.2.3. **Standard Solution:** 1.2 mg/ml of USP Amoxicillin RS in Buffer. [NOTE-Use this solution within 6 h.]

4.2.4. **Sample solution:** Remove, as completely as possible, the contents of NLT 20 Capsules. Mix the combined contents, and transfer a quantity, equivalent to 200 mg of anhydrous amoxicillin, to a 200-ml volumetric flask. Add Buffer to volume. Sonicate if necessary to ensure complete dissolution. [NOTE-Use this solution within 6 h.]

4.3. CHROMATOGRAPHIC CONDITIONS

4.3.1. **Column:** XBridge C 18, 4-mm x 25-cm; 10-µm packing or equivalent

4.3.2. **Flow Rate:** 1.5 mL/min

4.3.3. **Detector:** 230 nm

4.3.4. **Injection Volume:** 10 µL

4.4. SYSTEM SUITABILITY

4.4.1. **Sample:** Standard Solution

4.4.2. **Suitability Requirements:**

4.4.2.1. Tailing Factor NMT 2.5

4.4.2.2. The peak areas five consecutive injections of sample have a %RSD NMT 2.0%

4.5. ANALYSIS

4.5.1. **Samples:** Standard solution and Sample solution

4.5.2. **Calculations:** Calculate the percentage of the labeled amount of Amoxicillin (C16H19N3O5S) in the portion of Capsules taken:

Result = (ru/rs) x (Cs/Cu) x P x F x 100

ru = peak response from the Sample solution

rs = peak response from the Standard solution

Cs = concentration of USP Amoxicillin RS in the Standard solution (mg/ml)

Cu = nominal concentration of Amoxicillin in the Sample solution (mg/ml)

P = potency of Amoxicillin in USP Amoxicillin RS (µg/mg)

F = conversion factor, 0.001 mg/µg

4.6. ACCEPTANCE CRITERIA: 90.0%-120.0%
